# Genome-wide identification, splicing, and expression analysis of the myosin gene family in maize (*Zea mays*)

**DOI:** 10.1093/jxb/ert437

**Published:** 2013-12-21

**Authors:** Guifeng Wang, Mingyu Zhong, Jiajia Wang, Jushan Zhang, Yuanping Tang, Gang Wang, Rentao Song

**Affiliations:** Shanghai Key Laboratory of Bio-Energy Crops, School of Life Sciences, Shanghai University, No. 333 Nanchen Road, Shanghai, PR China

**Keywords:** Alternative splicing, evolution, expression pattern, headless myosin, maize, myosin.

## Abstract

The actin-based myosin system is essential for the organization and dynamics of the endomembrane system and transport network in plant cells. Plants harbour two unique myosin groups, class VIII and class XI, and the latter is structurally and functionally analogous to the animal and fungal class V myosin. Little is known about myosins in grass, even though grass includes several agronomically important cereal crops. Here, we identified 14 myosin genes from the genome of maize (*Zea mays*). The relatively larger sizes of maize myosin genes are due to their much longer introns, which are abundant in transposable elements. Phylogenetic analysis indicated that maize myosin genes could be classified into class VIII and class XI, with three and 11 members, respectively. Apart from subgroup XI-F, the remaining subgroups were duplicated at least in one analysed lineage, and the duplication events occurred more extensively in *Arabidopsis* than in maize. Only two pairs of maize myosins were generated from segmental duplication. Expression analysis revealed that most maize myosin genes were expressed universally, whereas a few members (*XI-1*, *-6*, and *-11*) showed an anther-specific pattern, and many underwent extensive alternative splicing. We also found a short transcript at the *O1* locus, which conceptually encoded a headless myosin that most likely functions at the transcriptional level rather than via a dominant-negative mechanism at the translational level. Together, these data provide significant insights into the evolutionary and functional characterization of maize myosin genes that could transfer to the identification and application of homologous myosins of other grasses.

## Introduction

In eukaryotic cells, motor proteins use the energy released from ATP hydrolysis to transport various intracellular cargos, including membranous organelles, protein complexes, and mRNAs, along tracks of cytoskeletal polymers (Lee and Liu, 2004). Of these systems, the actin–myosin system is required for the organization and dynamics of the endomembrane system and transport network in plant cells.

Plants harbour two unique myosin groups: class VIII and class XI. The latter is structurally analogous to class V in metazoans and fungi and contains a dilute domain in the globular tail (Li and Nebenfuhr, 2008). Class V myosin is a processive motor that is responsible for organelle and vesicle transport, and partitioning during cell division, mitotic spindle positioning, mRNA localization, and the establishment of cell polarity (Hammer and Sellers, 2012). Following the first cloned *Arabidopsis* myosin gene *ATM1* (Knight and Kendrick-Jones, 1993), many advances have been achieved in understanding plant myosin function over the past decade.

In *Arabidopsis*, the myosin family contains 17 genes, including 13 class XI (*XI-A*, *-B*, *-C*, *-D*, *-E*, *-F*, *-G*, *-H*, *-I*, *-J*, and *-K*, *XI-1*/*MYA1*, and *XI-2*/*MYA2*) and four class VIII (*VIII-1*/*ATM1*, *VIII-2*/*ATM2*, *VIII-A*, and *VIII-B*) members (Reddy and Day, 2001). Immunolocalization studies have indicated that ATM1 appears to be localized in plasmodesmata in cress and maize root cells, suggesting possible roles in cell-plate maturation and actin cable re-establishment (Reichelt *et al.*, 1999). Using transient expression of an ATM1 tail–green fluorescent protein fusion protein in tobacco, it was found that the signals co-localized with plasmodesmata and with the endosomal tracer FM4-64, implying a role of ATM1 in endocytosis (Golomb *et al.*, 2008). However, ATM2 could bind to early endosomes and mediate transportation through the endocytic pathway in concert with ATM1 (Golomb *et al.*, 2008; Sattarzadeh *et al.*, 2008). Ectopic expression of the tail domains of tobacco class VIII myosins but not those of class XI could inhibit the plasmodesmatal localization of the Hsp70 homologue of beet yellows virus (Avisar *et al.*, 2008a). In the moss *Physcomitrella patens*, the class VIII quintuple mutant Δmyo8ABCDE is smaller, produces more side branches, and forms gametophores earlier than the wild type; it also generates protonemal patterning defects in the absence of nutrient medium (Wu *et al.*, 2011). Thus, myosin VIII proteins are associated with endocytosis, cytokinesis, plasmodesmal function, and moss protonemal patterning.

In contrast, class XI contains a large number of members that originated from a burst of gene duplication. For example, *Arabidopsis* has 13 class XI genes with remarkable diversification. Immunolocalization studies have shown that MYA2 localizes on peroxisomes in epidermal and guard cells of *Arabidopsis* leaves in an actin-dependent manner (Hashimoto *et al.*, 2005), and that overexpression of the tail domains of MYA1, MYA2, XI-C, XI-E, XI-I, and XI-K impair the motility of peroxisomes, Golgi bodies, and mitochondria in tobacco leaves (Avisar *et al.*, 2008b, 2009; Sparkes *et al.*, 2008; Sattarzadeh *et al.*, 2011). Additionally, XI-K is physically associated with the endoplasmic reticulum (ER) and is required for its movement and remodelling (Sparkes *et al.*, 2009; Ueda *et al.*, 2010). An *in vitro* assay in tobacco BY2 cells indicated that myosin XI is also involved in tubular ER formation (Yokota *et al.*, 2011). Recently, it was found that the functional, full-length XI-K–yellow fluorescent protein (YFP) fusion protein was associated primarily with endomembrane vesicles trafficking along F-actin instead of with larger organelles (Peremyslov *et al.*, 2012).

However, functional dissection of the class XI myosins has largely been hindered owing to the redundancy of different paralogous genes. Only two (*XIK* and *MYA2*) of all 13 *Arabidopsis* class XI myosin mutants exhibit detectable phenotypes such as shorter root hairs under normal growth conditions (Peremyslov *et al.*, 2008). Double mutants of *Arabidopsis* class XI myosin pairs indicate overlapping and additive effects for *XI-K*, *XI-B*, and *MYA2* on root hair elongation (Prokhnevsky *et al.*, 2008). Additionally, the *xik mya2* mutant was stunted with reduced fecundity. Moreover, triple and quadruple mutants exhibited defects in cells undergoing polarized elongation and diffuse growth (Peremyslov *et al.*, 2010). Simultaneous silencing of moss myosin *XIA* and *XIB* resulted in severely stunted plants that were composed of small, rounded cells (Vidali *et al.*, 2010). Together, these data indicate that class XI myosins are involved in cytoplasmic streaming, organelle motility, and remodelling, and in plant growth and development.

In Poaceae, only limited progress has been achieved in myosin research, although grasses includes several agronomically important cereal crops with available whole-genome sequences. Twelve, seven, and nine class XI myosin genes exist in rice, sorghum, and brachypodium, respectively, and all contain two class VIII myosin genes (Jiang and Ramachandran, 2004; Peremyslov *et al.*, 2011). Rice myosin *XI-B* is required for normal pollen development by localizing its protein in a photoperiod-sensitive manner (Jiang *et al.*, 2007). Three myosin genes have been obtained in maize, and one of the XI members is associated with mitochondria, plastids, and the molecular chaperone subunit TCP-1α (Liu *et al.*, 2001; Wang and Pesacreta, 2004). Previously, we functionally characterized *opaque1*, which encodes a myosin XI protein and influences protein body assembly by affecting ER morphology and motility (Wang *et al.*, 2012a). Here, we employed bioinformatics and publicly available data to identify and analyse maize myosin genes at the genome-wide scale. These findings will provide an important blueprint for future maize myosin functional characterization that will transfer to other grass species for agricultural trait improvement, such as for *Opaque1*.

## Materials and methods

### Plant material

Maize inbred-line W22 plants were cultivated in the field at the campus of Shanghai University. Tissues (root, stem, the third leaf, silk, sheath, husk, tassel, and ear) were obtained from at least three healthy plants at the V12 stage as described previously (Wang *et al.*, 2010), and developing kernels were collected at 3, 9, 15, 21, 27 and 33 d after pollination. Tobacco (*Nicotiana benthamiana*) plants were grown in a greenhouse under a 16⁄ 8h day/night regime at a temperature of 20–25 °C (Wang *et al.*, 2011). Maize callus was induced using F1 seeds of the hybrid Hi II line (Hi II pA×Hi II pB) and grown and maintained in an improved N6 medium.

### Isolation and analysis of maize myosin genes

First, the conserved amino acids of the myosin head ATPase domain (Pfam: PF00063) and IQ motif (Pfam: PF00612) were used to search the maize genome database MaizeGDB (http://www.maizegdb.org/). Secondly, all *Arabidopsis* and rice myosin protein sequences were used as query sequences to search against the maize genome database and National Center for Biotechnology Information (NCBI) using the BLASTP program. The retrieved sequences were then assembled to remove redundancy. The Pfam (http://pfam.sanger.ac.uk/search) and SMART (http://smart.embl-heidelberg.de/) databases were used to confirm each predicted maize myosin sequence. For misannotated or split myosins, reverse transcription (RT)-PCR was used to combine the separated cDNA fragments with the primers described in Supplementary Table S1 (at *JXB* online).

### Gene model and splicing analysis of maize myosin genes

The information for annotated maize myosin genes, including accession number, chromosomal location, open reading frame (ORF) length and exon–intron structure, were retrieved directly from the B73 maize sequencing database (http://www.maizesequence.org/index.html), and the exon–intron organization of our filled, complete myosins was identified in the maize sequence database using the BLASTN program and constructed using the DNAMAN software. RepeatMasker searching was used to identify repetitive sequences that were present in large introns (>1kb) (Tarailo-Graovac and Chen, 2009). Maize RNA-seq transcriptome data were downloaded from the NCBI Short Read Archive (accession numbers SRX105522, SRX105660, SRX058602, SRX058603, SRX058601, SRX058608, and SRP006965; http://www.ncbi.nlm.nih.gov/sra). RNA-seq reads were mapped to the maize genome assemblies using the TopHat 2.0.9 software (http://tophat.cbcb.umd.edu/; Trapnell *et al.*, 2009).

### Phylogenetic analysis

The amino acid sequences of maize myosins, along with those of *Arabidopsis thaliana*, *Oryza sativa*, *Sorghum bicolor* and *Saccharomyces cerevisiae*, were submitted to the ClustalW program at the BCM search launcher (Baylor College of Medicine, Houston, TX) using their default settings (pairwise alignment options: gap opening penalty 10, gap extension penalty 0.1; multiple alignment options: gap opening penalty 10, gap extension penalty 0.2, gap distance 5, no end gaps and protein weight matrix using Gonnet) for multiple protein alignment. Based on the aligned protein sequences, the phylogenetic tree was constructed using the MEGA5.0 program (http://www.megasoftware.net/) and the neighbour-joining method, and the bootstrap test was carried out with 1000 replicates.

### Chromosomal distribution and myosin gene duplication in maize

Based on a previously constructed syntenic map of maize, rice, and sorghum (Schnable *et al.*, 2009), maize myosins were mapped on chromosomes by identifying their physical chromosome position, which was provided in the maize sequence database. Gene duplication events of maize myosin genes were investigated according to block pairs, as described previously (Wei *et al.*, 2007).

### RNA extraction and RT-PCR

Immature kernel RNA was isolated using a previously described protocol (Wang *et al.*, 2012b), and RNA was extracted from tissues using TRIzol RNA extraction reagent (Tiangen). The residual DNA was removed by RNase-free DNase I (Takara) treatment, and approximately 2 μg of total RNA from each sample was reverse transcribed to cDNA using RevertAid H Minus Reverse Transcriptase (Thermo Scientific). Semi-quantitative PCR was performed using a protocol described previously (Wang *et al.*, 2010). PCR primers were designed using the Primer Premier 5.0 program (Premier Biosoft) (Supplementary Table S1). To determine the transcription initiation site of *Opaque1*, 5′ RNA ligase-mediated rapid amplification of cDNA ends (RLM-RACE) was performed using a First Choice RLM-RACE kit (Ambion) according to the manufacturer’s protocol. Maize public expression datasets were obtained from the Plant Expression Database (PLEXdb; http://www.plexdb.org/) and a heat map was created using the pheatmap (Pretty Heatmaps) package (R version 3.0.2, pheatmap version, 0.7.7; R Core Team, Vienna, Austria).

### Subcellular localization

First, the enhanced YFP (eYFP) coding sequence was amplified from pB7WGY2.0 and inserted into pBI121 using the *Eco*RI–*Sal*I and *Xho*I sites. The O1-head-IQ and O1-head cDNA fragments were amplified from maize kernel and inserted into the reconstructed pBI121 vector using the *Eco*RI and *Sal*I sites, respectively. A transient expression assay was performed using a previously described method (Wang *et al.*, 2012a; Gao *et al.*, 2013), and images were obtained using a combination of 514nm laser excitation and 530–580nm long-pass emission filters using a Zeiss confocal microscope LSM 710 (Carl Zeiss). All images were analysed using Image J software (National Institutes of Health).

### Analyses of headless myosin gene expression

A DNA fragment containing the β-glucuronidase (GUS) coding region and nopaline synthase (NOS) terminator was obtained from pBI121 and inserted into pUC18 using the *Sma*I and *Eco*RI sites. The P1 and P2 fragments were amplified from genomic DNA of B73 using the primers in Supplementary Table S1 and cloned into the above pUC18 harbouring GUS and the NOS terminator using the *Xba*I and *Sma*I sites. The resulting plasmids (1 µg) were bombarded into maize calli with a Biolistic PDS-1000/He Gene Gun System (Bio-Rad) and GUS activity was investigated by incubating the samples in a 5-bromo-4-chloro-3-indolyl β-glucuronide-containing standard histochemical solution overnight at 37 °C.

## Results

### The maize myosin gene family has 14 members

With the complete maize genome sequence available (Schnable *et al.*, 2009), BLASTP and TBLASTN searches were performed in MaizeGDB, using the head domain and full-length amino acid sequences of the rice (*O. sativa*) and *Arabidopsis* myosin proteins. We first retrieved 22 sequences encoding myosin homologues (Supplementary Table S2 at *JXB* online). Fourteen of the sequences were incomplete because they contained only head or tail domains. Of these sequences, six pairs contained separated myosin head and dilute domains at the approximate positions on the chromosomes, which indicated that these coupled sequences were most likely split from a complete myosin gene.

RT-PCR was used to fill the gaps between the coupled sequences, and we successfully obtained the missing cDNA sequences of the six incomplete myosin genes. Therefore, 14 complete myosin genes were identified in the maize genome (Table 1), including the previously reported *Opaque1* (Wang *et al.*, 2012a), ZMM1, ZMM2, and ZMM3 (Liu *et al.*, 2001). This number was comparable to that of its monocot relatives *Sorghum* (12) and rice (14), but was less than that in *Arabidopsis* (17). The genomic region of the maize myosin genes ranged from 8.218 to 85.126kb and encoded proteins of 990–2641 aa. Noticeably, the average size of maize myosins was 32.918kb (class VIII, 12.177kb; class XI, 38.575kb), which was much larger than those in *Sorghum* (16.887kb), rice (16.595kb), and *Arabidopsis* (8.981kb).

**Table 1.** Myosins identified from the completed maize genome sequence

Myosin name/new nomenclature^a^	Chromosome				Length (kb)	Exon	Gene accession no.	No. of aa	MW (kDa)	pI	Evidence	***Arabidopsis ***best hit
	No.	Position (bp)		Strand								
		From	To									
ZmVIII-1/ ZmMyo8A1	1	229190070	229205919	–	15.850	23	GRMZM2G113202	1191	133.472	8.53	Y(M3)	VIII-1
ZmVIII-2/ ZmMyo8A2	5	25983026	25994059	–	11.034	23	GRMZM2G139583	1194	134.072	8.50	Y	VIII-1
ZmVIII-3/ ZmMyo8B	7	157571319	157580965	–	9.647	24	GRMZM2G057380	1238	139.825	9.13	Y	VIII-2
ZmXI-1/ ZmMyo11A2	1	300108722	300149560	–	40.839	38	GRMZM2G471108	1506	170.675	7.70	Y	XI-B
ZmXI-2/ ZmMyo11E2^*a*^	3	203463334	203507842	+	44.509	52	KF493895	2641	299.535	5.93	Y	XI-K
ZmXI-3/ ZmMyo11E3	3	220883061	220902116	–	19.056	39	AC155377.1_FG001	1529	173.465	8.53	Y(M1)	XI-K
ZmXI-4/ ZmMyo11G1	4	176870473	176897290	–	26.818	38	GRMZM2G449909	1520	173.204	8.85	Y(O1)	XI-I
ZmXI-5/ ZmMyo11H	5	7359267	7367484	–	8.218	34	GRMZM2G460396	990	113.532	8.01	Y	XI-F
ZmXI-6/ ZmMyo11G2	5	36445589	36530714	+	85.126	38	KF493897	1522	174.373	8.83	Y	XI-I
ZmXI-7/ ZmMyo11A3	5	215701287	215714449	+	13.163	39?	KF493892	1554	175.882	8.48	Y(M2)	XI-B
ZmXI-8/ ZmMyo11E1	6	161483674	161547583	+	63.910	45	KF493894	1973	224.613	6.87	Y	XI-K
ZmXI-9/ ZmMyo11A1	7	36208341	36261130	+	52.790	39?	KF493893	1506	171.027	8.04	Y	XI-B
ZmXI-10/ ZmMyo11E2b	8	154701271	154761121	–	59.851	45?	KF493896	1880	213.827	7.07	Y	XI-K
ZmXI-11/ ZmMyo11C	9	4288567	4298608	–	10.042	39	GRMZM2G435294	1529	173.418	8.92	Y	XI-E

^*a*^ The new nomenclature of myosin genes proposed by Madison and Nebenfuhr (2013).

### Maize myosin genes possess typical domains but complicated intron–exon organization

**Fig. 1.**
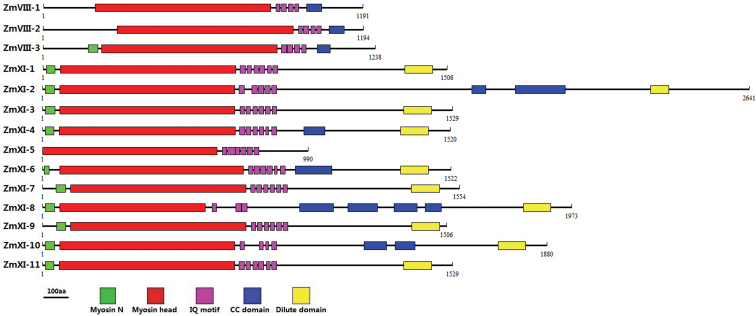
Schematic diagram of maize myosins. The putative domains or motifs were identified using the Pfam and SMART databases with the default parameters. Myosin N, N-terminal SH3-like; Myosin head, large ATPase; IQ, putative calmdulin-binding motif; CC, coiled coil; DIL, dilute. Bar, 100 aa. (This figure is available in colour at *JXB* online.)

The Pfam (Punta *et al.*, 2012) and SMART (Letunic *et al.*, 2012) databases were used to identify the putative domains that were present in the 14 complete maize myosins (Fig. 1, Supplementary Table S3 at *JXB* online). The myosins all contained a large, ATPase motor domain and several IQ motifs, which were used for ATP hydrolysis and binding calmdulin, respectively. Except for the 990 aa member (GRMZM2G460396), maize myosins could apparently be divided into two classes (VIII and XI) according to the remaining domains. Compared with class VIII, the class XI myosins were much longer and had an N-terminal SH3-like domain and a tail dilute domain. Similar to that in *Arabidopsis* and rice, the maize myosin head domain was located more towards the N terminus in class XI than in class VIII. Moreover, the class VIII myosins contained a coiled-coil domain and four IQ motifs, whereas a variable number of both domains were present in class XI members.

**Fig. 2.**
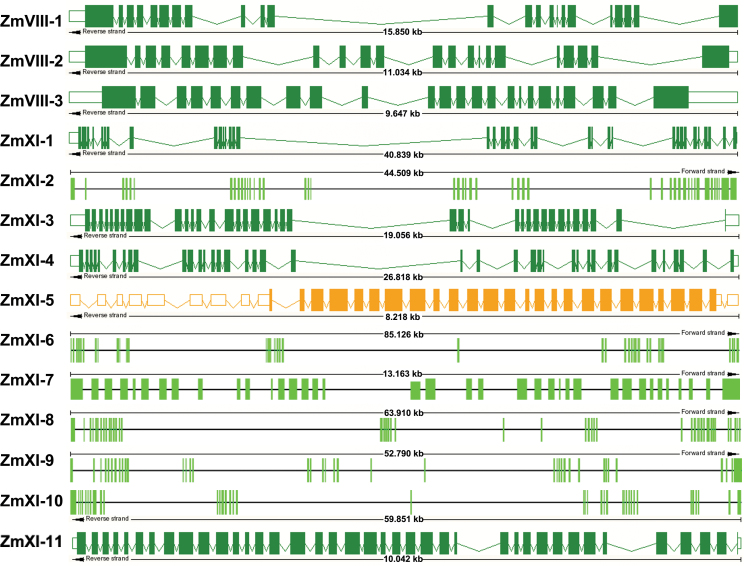
Exon–intron organization of the maize myosin genes. Boxes, exons; lines, introns. The dark-green and orange data are from the maize sequencing database (http://www.maizesequence.org/index.html), and the six light-green gene models come from this study. (This figure is available in colour at *JXB* online.)

The intron–exon structures of the maize myosin genes were determined by comparison of the cDNA with genomic sequences. The results revealed that maize myosin genes consisted of 23 (*ZmVIII-1* and *ZmVIII-2*) to 52 (*ZmXI-2*) exons, and the sizes varied from 14 to 1199bp (Fig. 2, Table 1 and Supplementary Table S4 at *JXB* online). The class VIII myosin genes contained 23–24 exons, whereas class XI genes harboured 38–52 exons. Noticeably, the length of most exons appeared to be conserved in the same order in the different maize myosin genes.

Compared with the conserved exon sizes in the same order, the introns were more divergent in length (57bp to 22.499kb) and caused the large sizes of the maize myosin genes. RepeatMasker searching was used to identify repetitive sequences that were present in large introns (>1kb) (Tarailo-Graovac and Chen, 2009). As shown in Supplementary Table S5 (at *JXB* online), retroelements (L1/CIN4, Ty1/Copia, SINEs, RTE/Bov-B, and Gypsy/DIRS) and DNA transposons (hobo-Activator, Tourist/Harbinge, and Tc1-IS630-Pogo) were abundant in maize myosin introns. This finding revealed that transposable elements play an essential role in maize myosin size increase and intron–exon organization.

### Maize myosins are less duplicated than those of *Arabidopsis*

**Fig. 3.**
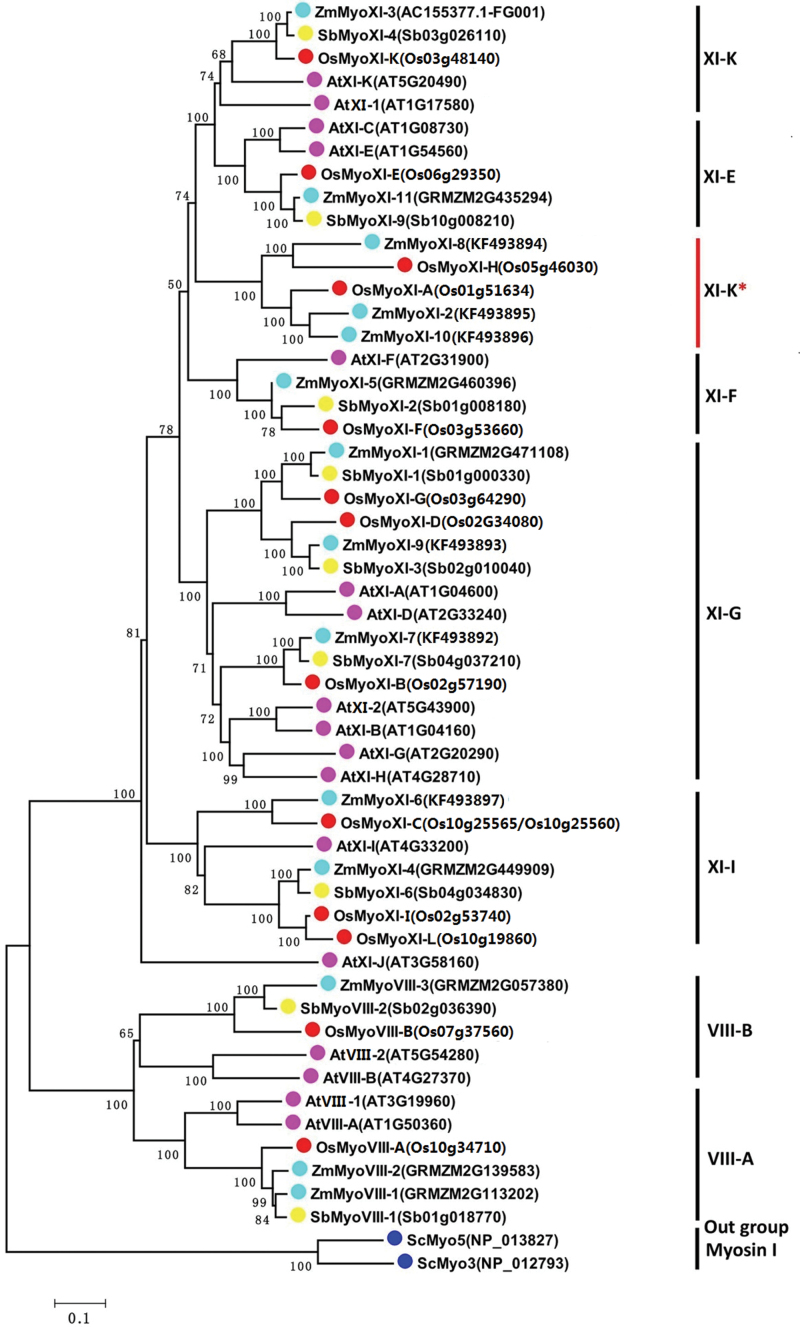
Phylogenetic relationships between the entire myosins of maize, rice, sorghum, and *Arabidopsis*. The phylogenetic tree was constructed using MEGA 5.0 software, and yeast class I myosins were used as outgroups (see Materials and methods). At, *Arabidopsis thaliana*; Os, *Oryza sativa*; Sb, *Sorghum bicolor*; Sc, Saccharomyces cerevisiae; Zm, Zea mays.

The available myosin gene family in *Arabidopsis*, rice, and sorghum allowed us to investigate the evolutionary relationship between dicot and monocot myosin proteins. A neighbour-joining tree was constructed using the full-length protein sequences of 17 *Arabidopsis*, 13 rice, 9 sorghum, and 14 maize myosins, with two yeast class I myosins as an outgroup (Fig. 3). The results indicated that these myosins were divided distinctly into two groups, class VIII and class XI, which supports the suggestion that two myosin ancestors existed *in planta* before lineage-specific expansions (Peremyslov *et al.*, 2011). Eleven myosins from maize, 13 from *Arabidopsis*, 11 from rice and seven from sorghum in class XI were found, whereas the smaller VIII group contained three maize, four *Arabidopsis*, and two rice and sorghum members. This result was generally consistent with the conclusion that was made previously using myosin motor domains (Peremyslov *et al.*, 2011). The class VIII myosins were divided into two distinct subgroups (VIII-A and VIII-B), and class XI was split into the I, G, F, K, and E subdivisions (Peremyslov *et al.*, 2011; Wang *et al.*, 2012a). Interestingly, *Arabidopsis* XI-J was grouped alone into a clade without any close homologues.

It was clearly shown that class VIII myosins had undergone a single-gene duplication but that a burst of duplication occurred in class XI (Fig. 3). Two paralogous branches were formed within subclasses VIII-A and VIII-B in the dicot *Arabidopsis* compared with only one in monocots, excepting for the two in maize VIII-B. The largest subgroup, XI-G, contained six members in *Arabidopsis* but only three in the monocots rice, sorghum, and maize. Moreover, two paralogues were found within XI-K and XI-E in *Arabidopsis*, compared with only one in monocots. Consistently, subgroup XI-F was the only subdivision that was not duplicated in any of the plant lineages (Peremyslov *et al.*, 2011). Noticeably, three maize and two rice myosins comprised a separate clade that was previously designated XI-K (Jiang and Ramachandran, 2004).

### Chromosomal localization and myosin gene duplication in grass

**Fig. 4.**
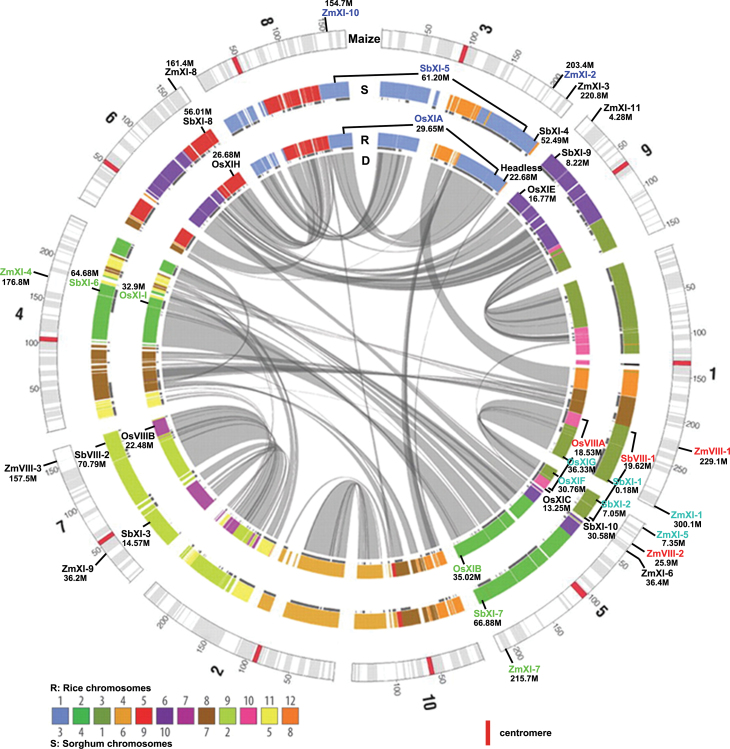
Chromosomal localization and segmental duplication of myosin genes in the maize genome (adapted from Schnable *et al.*, 2009). S and R represent the syntenic blocks between maize and sorghum and rice, respectively. D indicates oriented homologous sites of duplicated gene blocks within maize. The coupled colours in the maize myosins represent genes that were located in the same block. (This figure is available in colour at *JXB* online.)

To investigate the chromosomal distribution of the myosin family in maize, the loci were determined by directly identifying their physical positions provided in MaizeGDB. As shown in Fig. 4, the 14 myosins were mapped on eight out of the 10 maize chromosomes, excluding chromosomes 2 and 10. The largest number of myosin members (four) was present on chromosome 5, followed by two genes each on chromosomes 1, 3, and 7. The remaining chromosomes (4, 6, 8, and 9) each had a unique myosin.

Maize originated from an ancient allotetraploid and has undergone several rounds of whole-genome duplication events during its gene evolution (Wei *et al.*, 2007; Schnable *et al.*, 2009). Among these maize myosin genes, four sister pairs (*ZmVIII-1* and *ZmVIII-2*, *ZmXI-1* and *ZmXI-5*, *ZmXI-2* and *ZmXI-10*, and *ZmXI-4* and *ZmXI-7*) appeared to be generated from segmental duplication events due to their position on the same duplicated gene blocks within maize (Fig. 4). To confirm this possibility, we further analysed myosin gene evolution among maize, rice, and sorghum (Table 2). Thirteen of 14 maize myosins had collinear genes in rice (the exception was *ZmXI-9*), while all had syntenic members in sorghum. Two of the sister pairs (*ZmVIII-1* and *ZmVIII-2*, and *ZmXI-2* and *ZmXI-10*) each had a unique, syntenic myosin in rice and sorghum, which indicated that they were generated from segmental duplication after the divergence between maize and its relatives (rice and sorghum). The remaining two pairs (*ZmXI-1* and *ZmXI-5*, and *ZmXI-4* and *ZmXI-7*) were each located on the same duplicated blocks, but coupled, syntenic myosins were found in rice and sorghum. It is possible that the ancestor of grasses already contained two myosin genes at this region and that one copy of each duplicate myosin pair was lost at the duplicated blocks after the whole-genome duplication in maize (Schnable *et al.*, 2011). In addition, three rice myosin genes (*OsXI-D*, *OsXI-K*, and *OsXI-L*) had no syntenic member in maize or sorghum. *OsXI-L* was most likely duplicated from *OsXI-I*, whereas *OsXI-D* and *OsXI-K* appeared to be duplicated or redistributed from the original loci to the current positions, and the original gene was partially or fully lost (Salse *et al.*, 2008).

**Table 2.** *Analysis of gene duplication in maize myosins*The light grey and light and medium brown shading represent maize, rice, and sorghum myosin genes, respectively (from top to bottom); the other coupled shading represents the myosin genes in the same duplication block in maize.

Maize myosin	Physical block^*a*^	Chr	Block pairs^b^	Rice syntenic region	Physical position^c^	Chr	Sorghum syntenic region	Physical position^*c*^	Chr
ZmVIII-1	46	1	10La	Os10g34710 (OsVIII-A/B^***d***^)	18.53	10	Sb01g018770 (SbVIII-1)	19.62	1
ZmVIII-2	212	5							
ZmVIII-3	322	7	7L	Os07g37560 (OsVIII-B/A^***d***^)	22.48	7	Sb02g036390 (SbVIII-2)	70.79	2
ZmXI-1	67	1	3L	Os03g64290 (OsXI-G)	36.33	3	Sb01g000330 (SbXI-1)	0.18	1
ZmXI-5	206	5		Os03g53660 (OsXI-F/E^***d***^)	30.76		Sb01g008180 (SbXI-2)	7.05	
ZmXI-2	143	3	1L	Os01g51630, Os01g51632, Os01g51634 (OsXI-A)	29.65^***e***^	1	Sb03g032760, Sb03g032770 (SbXI-5)	61.20^***e***^	3
ZmXI-10	359	8							
ZmXI-3	150	3	1L	Os01g40200 (Headless)	22.68	1	Sb03g026110 (SbXI-4)	52.49	3
-	-	-	-	Os03g48140 (OsXI-K/F^***d***^)	27.37	3	-	-	-
ZmXI-4	182	4	2Lb	Os02g53740 (OsXI-I/C^***d***^)	32.90	2	Sb04g034830 (SbXI-6)	64.68	4
ZmXI-7	254	5		Os02g57190 (OsXI-B)	35.02		Sb04g037210 (SbXI-7)	66.88	
ZmXI-6	217	5	10La	Os10g25565 Os10g25560 (OsXI-C/K^**d**^)	13.25^***e***^	10	Sb01g023160, Sb01g023170 (SbXI-10)	30.58^***e***^	1
ZmXI-8	287	6	5L	Os05g46030 (OsXI-H)	26.68	5	Sb09g026840, Sb09g026850 (SbXI-8)	56.01^***e***^	9
ZmXI-9	300	7	7S	-	-	-	Sb02g010040 (SbXI-3)	14.57	2
-	-	-	-	Os02g34080 (OsXI-D)	20.36	2	-	-	-
ZmXI-11	368	9	6S	Os06g29350 (OsXI-E/J^***d***^)	16.77	6	Sb10g008210 (SbXI-9)	8.22	10
-	-	-	-	Os10g19860 (OsXI-L)	9.90	10	-	-	-

^*a*^ Maize FPC contigs (http://www.genome.arizona.edu/fpc/maize/WebFPC/) have been numbered sequentially along each chromosome.

^*b*^ Regions on chromosomes that have contiguous contigs that are syntenic with rice as defined by SyMAP.

^*c*^ The physical position on each chromosome.

^*d*^ The former letter represents the rice myosin gene name that was proposed by this study; the latter indicates that proposed by Jiang and Ramachandran (2004).

^*e*^ The misannotated myosin genes.

### Expression pattern of maize myosin genes

**Fig. 5.**
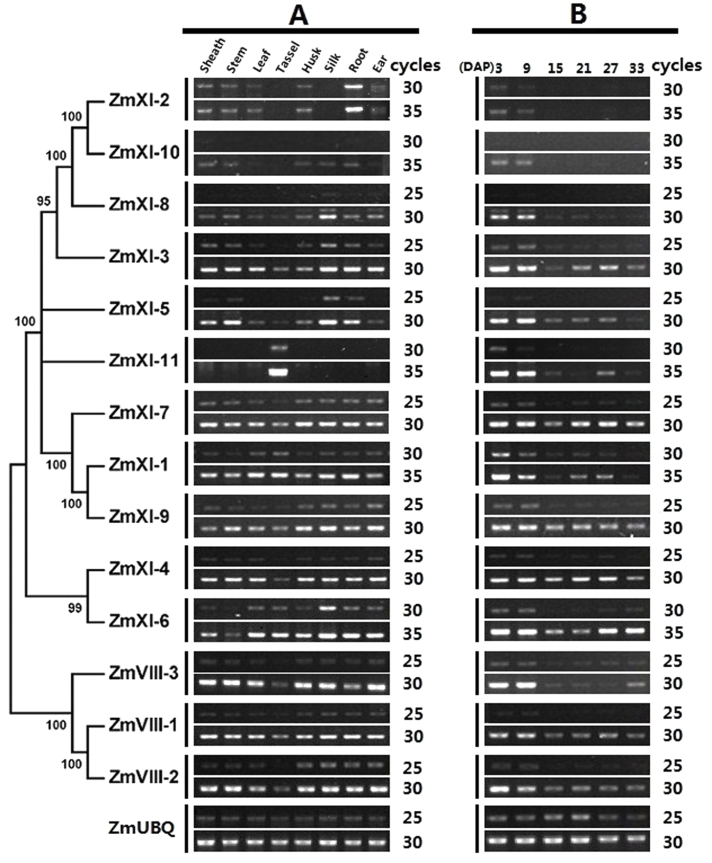

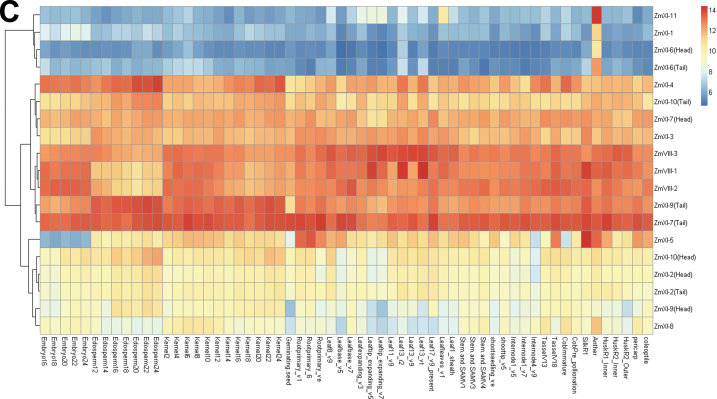
Expression patterns of maize myosin genes. (A) RNA expression levels of maize myosin genes in various tissues. (B) Expression profiles of myosin genes during maize kernel development. (C) Development- and anatomy-specific expression profiles of maize myosin genes. Public microarray data sets were obtained from PLEXdb. The genes are located on the right and the tissues are indicated at the bottom of each column. The colour bar represents the expression values. Head and tail represent the splicing cDNAs encoding myosin head and tail domains, respectively. (This figure is available in colour at *JXB* online.)

**Fig. 6.**
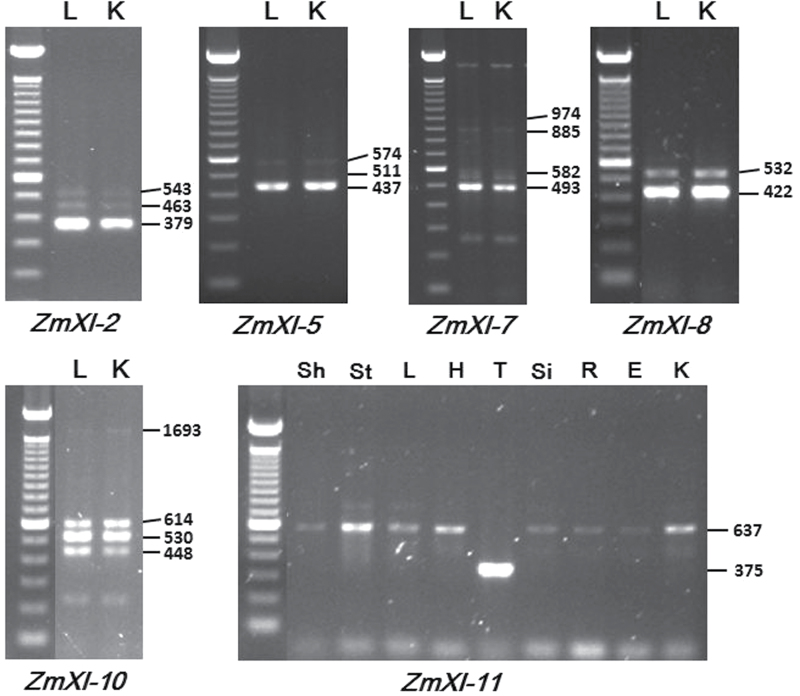
Alternative splicing of maize myosins. Leaf (L) and kernel (K) were used in RT-PCR with the primers listed in Supplementary Table S1. The numbers on the right indicate the lengths (bp) of the spliced transcripts. (This figure is available in colour at *JXB* online.)

To investigate the spatial patterns of myosin gene expression in maize, we detected their transcripts using RT-PCR in eight representative tissues, i.e. sheath, stem, leaf, tassel, husk, silk, root, and ear (Fig. 5A). The results revealed that the majority of maize myosin genes (excluding *ZmXI-2* and *ZmXI-10*) were expressed at varying levels in all tested tissues. The *ZmXI-2* transcripts were particularly abundant in root but absent in silk and tassel. In comparison, its duplicated sister, *ZmXI-10*, was expressed at low levels in all tested tissues and was undetectable in tassel. Three myosin XI genes, *ZmXI-3*, *ZmXI-5*, and *ZmXI-8*, exhibited high transcription levels in silk. Noticeably, *ZmXI-11* was highly expressed in tassel, whereas the remaining myosins were less abundant in tassel when compared with the other tissues. Interestingly, the short *ZmXI-11* transcript was unique to tassel, while the long, unspliced transcript was common in the remaining tissues (Fig. 6). Furthermore, the two duplicated myosin gene pairs shared similar expression patterns in all tissues. We also unravelled the expression profiles of the maize myosin genes during kernel development (Fig. 5B). Transcripts of all 14 maize myosins were detectable with dynamic patterns. Most were expressed preferentially at the early stage, and peaked approximately 3 and 9 d after pollination. *ZmXI-3*, *ZmXI-6*, and *ZmXI-7* exhibited a distinct pattern, with two expression peaks at the early and late stages of kernel development. The duplicated gene pairs also showed similar expression profiles.

We also investigated the expression levels of maize myosins in a broad range of tissues and organs of the inbred line B73 using a NimbleGen microarray (Sekhon *et al.*, 2011). For the currently misannotated full-length myosin genes, we used probes corresponding to their head and tail domain for further analysis. According to their expression levels, maize myosin genes could be clustered into three categories (Fig. 5C). The first category was composed of eight genes (*ZmVIII-1*, *-2* and *-3*; *ZmXI-3*, *-4*, *-7*, *-9-tail* and *-10-tail*) with high and ubiquitous expression levels, suggesting comprehensive roles in plant growth and development. All maize class VIII myosins belonged to this category; however, their biological functions remain to be elucidated. Five class XI myosins (*ZmXI-2 -5*, *-8*, *-9-head* and *-10-head*) formed the second category with intermediate and differential transcription levels. The third category included *ZmXI-1*, *-6* and *-11*, for which the expression levels were relatively low. However, all three genes were expressed preferentially in anther, especially *ZmXI-11* (Fig. 5C), implying that these myosins are required for anther and/or pollen growth and development.

### Alternative splicing is a common character of the maize myosin genes

Alternative splicing plays an essential role in regulating gene expression during growth and development, as well as in response to biotic and abiotic stresses (Stamm *et al.*, 2005; Syed *et al.*, 2012). RNA-seq analysis has revealed that more than 61% of intron-containing genes in *Arabidopsis* undergo alternative splicing under normal growth conditions (Marquez *et al.*, 2012). Due to their large sizes and the complicated gene models of myosins, we could speculate that maize myosin genes underwent extensive alternative splicing events. We then detected the splicing transcripts of the full-length cDNA for each maize myosin gene in leaf and kernel in two randomly selected windows. Six out of the 14 myosin genes had splicing transcripts in the given cDNA fragment in the two samples (Fig. 6 and Supplementary Fig. 1 at *JXB* online). Of these genes, four (*ZmXI-2*, *-*5, *-7* and *-10*) possessed at least three splicing transcripts, while the remaining had two transcripts. Interestingly, *ZmXI-11* was spliced in a tissue-dependent manner. A 375bp cDNA fragment of *ZmXI-11* was unique to tassel, while the others had an unspliced, 637bp transcript (Fig. 6). It is unknown whether this alternative splicing affects the subsequent translation. In the case of the maize *ZmXI-11* gene, the spliced transcript encoded a complete myosin (1529 aa), whereas the unspliced transcript generated a truncated myosin of 1454 aa in length that lacked a portion of the DIL domain. Together with its preferential expression pattern, this finding suggests that *ZmXI-11* most likely plays an essential role in microsporogenesis.

**Fig. 7.**
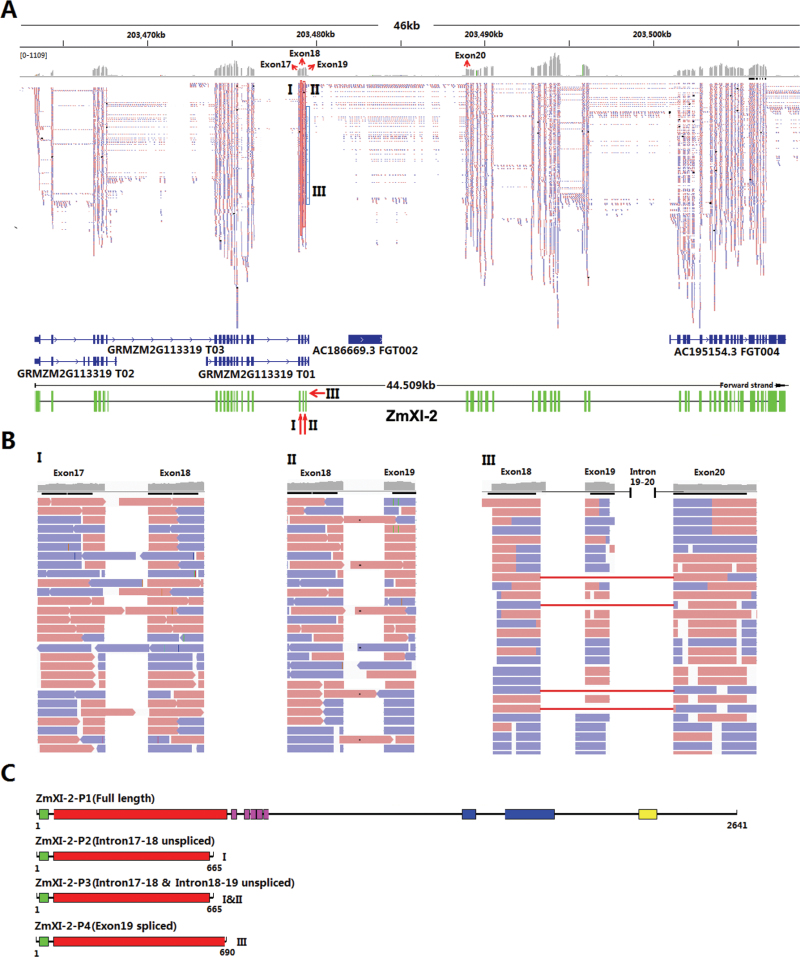
Transcript evidence and splicing for maize myosin *ZmXI-2*. RNA-seq reads were mapped to the maize genome assemblies using TopHat 2.0.9 software. (A) RNA-seq reads distribution and the empirical gene models of *ZmXI-2*. A box and an arrow mark alternative splicing events. Reads coloured in red and blue represent the sense and antisense sequences, respectively. (B) Validation of splicing details of *ZmXI-2* detected in Fig. 6. I, intron 17–18 retention; II, intron 18–19 retention; III, exon 19 splicing. Intron 17–18 and Intron 18–19 unspliced correspond to the 543bp band in Fig. 6; intron 17–18 unspliced corresponds to the 463bp band. (C) Deduced protein models of ZmXI-2 and its splicing transcripts. All the splicing variants encode truncated proteins only having the myosin head domain.

To evaluate further the gene models for maize myosins, transcript evidence created using the high-throughput RNA-seq transcriptome data from developing kenels was compared with all publicly available myosin genomic and transcript data using the TopHat 2.0.9 software (Trapnell *et al.*, 2009). According to this method, we revised the gene models for 13 maize class VIII and class XI myosins, except for *ZmXI-11* with few reads in the RNA-seq data (Supplementary Fig. 2 at *JXB* online). In the case of *ZmXI-2*, the RNA-seq transcript evidence supported the results that *ZmXI-2* was a complete myosin gene (Fig. 7A) and our RT-PCR detected splicing transcripts did exist in the transcriptomic data (Fig. 7B). In addition, the RNA-seq data revealed a novel exon splicing event in annotated exon 19 (Fig. 7B). All these splicing variants resulted in truncated proteins that only harboured the myosin head domain (Fig. 7C).

### O1, a functional myosin, is responsible for ER streaming and morphology that ultimately affects endosperm texture

**Fig. 8.**
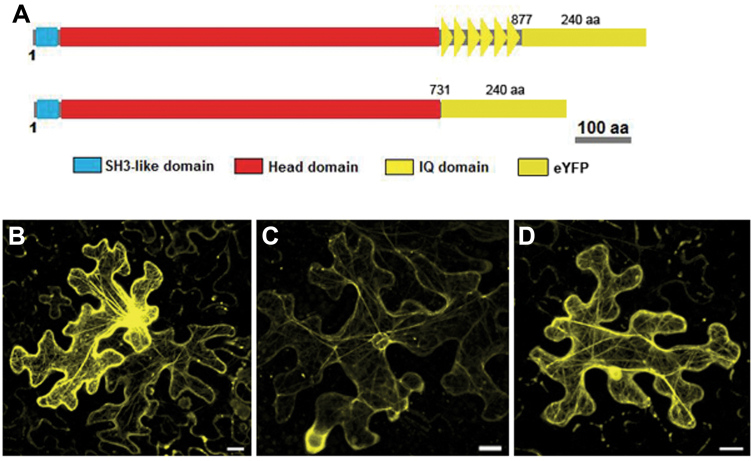
O1 is associated with actin filaments. (A) Schematic diagrams of the CaMV35S::O1(Head-IQ or Head)–eYFP vectors in which eYFP was fused at the C terminus. (B–D) Representative tobacco leaf pavement cells are shown by confocal microscopy 2 d after agroinfiltration. O1(Head-IQ) is present in actin filaments (B) and decorates the nucleus (C); the signals of the O1(Head) are similarly present in actin filaments (D). (This figure is available in colour at *JXB* online.)

Previously, we isolated the maize endosperm mutant *opaque1* (*o1*) using map-based cloning and found that it encoded a class XI myosin protein (Wang *et al.*, 2012a). *O1* was identical to *ZmXI-4*, and resulted in a transcript of 5131bp, encoding a 173kDa protein of 1520 aa. To study the association of *ZmXI-4*/*O1* with actin filaments, we transiently expressed the ZmXI-4 (Head-6IQ/Head)–eYFP reporters, which harboured the head domain with/without the IQ motif fused to eYFP at its C terminus (Fig. 8A), for direct, microscopic observation. The signal of the ZmXI-4 head–neck domain protein showed a clear pattern of actin filaments throughout the entire cell (Fig. 8B) that also decorated the nucleus (Fig. 8C). When only the ZmXI-4 head domain was expressed, the YFP signal was similarly associated with actin filaments (Fig. 8D), indicating that the head domain was essential for myosin binding to actin filaments and that the IQ motif had no effect on this process. Together with overexpression of the ZmXI-4/O1 tail domain significantly inhibiting ER streaming in tobacco cells and the phenotypes of the *opaque1* mutant, O1 was characterized as a functional myosin XI protein that was responsible for protein body assembly and endosperm texture determination (Wang *et al.*, 2012a).

**Fig. 9.**
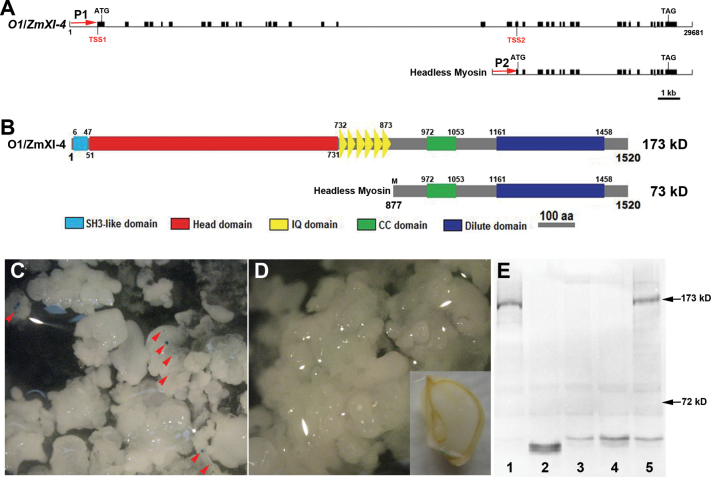
Identification and analysis of the headless derivative of *O1*. (A) A putative gene model of the maize headless myosin. TSS, transcription start site; P1 and P2, promoter regions of *O1* and the headless myosin. (B) Conserved domains present in the maize headless myosin, which lose the N-terminal SH3-like domain, head domain, and IQ motif. (C) GUS staining of the O1_pro_::GUS construct observed in the maize callus. Red arrowheads indicate the blue signals. (D) No activity is shown for the headless myosin promoter in the callus and developing kernel. (E) Immunoblot analysis of O1 and the headless myosin proteins in developing maize kernels at 20 d after pollination using an antibody described previously (Wang *et al.*, 2012). Lanes: 1, W22 inbred line; 2, o1-ref; 3, o1-N1478A; 4, o1-N1243; 5, the wild type. The arrows on the right indicate the expected protein weights of O1 and the headless myosin proteins. (This figure is available in colour at *JXB* online.)

We further explored whether functional myosin headless variants were present in the genome and their roles during plant growth and development. Apart from the longest 5131bp, mature transcript of *ZmXI-4*/*O1*, we also identified a 2380bp transcript that consisted of the second half of the longest transcript at the same locus (Fig. 9A) using two independent, 5′ RACE experiments (Supplementary Fig. 3 at *JXB* online). This short transcript contained an ORF of 1935bp that conceptually encoded a 73kDa headless myosin. Domain prediction analysis revealed that the deduced protein contained coiled-coil and DIL domains, and completely lost the N-terminal SH3-like domain, head domain, and IQ motif (Fig. 9B). To verify whether this headless myosin was a novel gene model or an alternative splicing variant of *O1*, the approximately 2kb genomic regions that were located upstream of the transcription start sites of *O1* (P1) and the headless myosin(P2) were fused to the GUS ORF. After bombardment into maize callus, substantial GUS staining was observed for the O1_pro_::GUS construct, while the headless myosin promoter showed no activity in the callus or developing kernel (Fig. 9C, D). Curiously, the predicted headless myosin protein was also undetectable in all samples using an immunoblot assay (Fig. 9E). This finding suggested that this short transcript was a headless derivative of *O1* and most likely functioned at the transcriptional level rather than in regulation of O1 activity via dominate-negative suppression *in vivo*.

## Discussion

Myosins are cytoskeletal molecular motors that use the energy released from ATP hydrolysis to move along actin filaments. In a genome-wide screen, we identified 14 complete myosin genes that harboured conserved domains in maize. A comparable number of myosin genes exists in maize (14), rice (14), and *Arabidopsis* (17), although the maize genome size (2300Mb) (Schnable *et al.*, 2009) is ~5.3 and ~18.4 times larger than those of rice (430Mb) (Burr, 2002) and *Arabidopsis* (125Mb) (Arabidopsis, 2000), respectively. This huge discrepancy might partially be compensated by the larger average size of the maize myosin genes, which are nearly two and four times longer than those in rice and *Arabidopsis*, respectively. Moreover, the exon sizes of the maize myosin genes are similar to those in rice and *Arabidopsis*, but maize myosin genes contain larger introns because of the insertion of transposable elements (Supplementary Table S5). Overall, transposable elements comprise approximately 85% of the B73 maize genome (Schnable *et al.*, 2009) and play important roles in genome organization and evolution (Fedoroff, 2012), as well as in gene-family expansion (Janousek *et al.*, 2013), which is most likely what is occurring in the maize myosin gene family.

Phylogenetic reconstruction analyses using the sequence information of several complete genomes of the green algae, mosses, and higher plants indicate that two types of myosin (VIII and XI) are present *in planta* (Avisar *et al.*, 2008b) and that three class VIII and 11 class XI myosin members are present in maize. In most cases, subgroups are duplicates of at least one lineage (Fig. 4). For example, subgroup VIII-A and VIII-B were duplicated after the separation between dicot *Arabidopsis* and monocots, while VIII-A was duplicated after the divergence between maize and its relatives. Prominently, only XI-F has not been duplicated within any lineage. The *Arabidopsis* T-DNA mutant of XI-F showed no discriminable phenotypes in vegetative growth (Peremyslov *et al.*, 2008), and some unexpected clades, such as *Arabidopsis* XI-J and the maize- and rice-specific XI-K, were found. Thus, the specific function of these myosin genes still requires further experimental elucidation.

Two rounds of whole-genome duplications have occurred during cereal genome evolution: the first occurred before the divergence of maize, rice, and sorghum, whereas the second took place in the specification of the maize lineage (Salse *et al.*, 2009). It appears that relatively more family members exist in maize than in other cereals; however, this is not the case for the maize myosin gene family (Fig. 5, Table 2). Overall, most of the myosins were present before the specification of maize, rice, and sorghum, but they were subjected to gene loss and redistribution within a specific lineage. For example, the rice sytenic gene (*OsXI-D*) of *ZmXI-9* was lost in the syntenic region 7S but was redistributed to a new locus in rice chromosome 2. Only two myosin pairs (*ZmVIII-1* and *ZmVIII-2*, and *ZmXI-2* and *ZmXI-10*) were segmentally duplicated after the divergence of maize and its relatives. Additionally, the *Arabidopsis* myosins have been amplified in most of the subgroups. Therefore, the events that triggered these lineage-specific duplications of the myosin genes and the subfunctionalization or neofunctionalization of their paralogous myosins following the duplications are becoming known.

Due to the global overlapping expression patterns of the *Arabidopsis* myosin genes (Peremyslov *et al.*, 2011), only two (*MYA2* and *XI-K*) of all 13 class XI myosin single mutants exhibited detectable defects under normal growth conditions (Peremyslov *et al.*, 2008), whereas their double, triple, and quadruple mutants displayed similar but more sever phenotypes when compared with the single mutants (Prokhnevsky *et al.*, 2008; Peremyslov *et al.*, 2010; Ueda *et al.*, 2010; Ojangu *et al.*, 2012). Transcripts of all myosin family members were detected in all tested tissues and organs of maize, although their abundance was considerably varied (Fig. 6). It has been shown that the global expression patterns of a fraction of orthologous genes are conserved in animals (Zheng-Bradley *et al.*, 2010) and plants (Davidson *et al.*, 2012). In the case of orthologous myosin genes in maize and *Arabidopsis*, most display similar expression patterns. For example, the *Arabidopsis* orthologues (*XI-C* and *XI-E*) of the maize anther-expressed *ZmXI-11* displayed extremely low levels in the entire plant but exclusively high levels in the stamen/anther (Peremyslov *et al.*, 2011), suggesting their important roles in pollen growth. In addition, the segmental duplicated maize myosin pairs shared correlated expression patterns when compared with other paralogues.

In humans, alternative splicing of myosin Va occurs in a region lying between the coiled-coil region of the IQ neck and the globular tail region in a tissue-specific manner (Hodi *et al.*, 2006; Wagner *et al.*, 2006; Roland *et al.*, 2009; Wu *et al.*, 2002), and usually three exons in this region are subject to alternative splicing. For example, exon B is required for the dynein light chain 2 (DLC2)–myosin Va interaction (Hodi *et al.*, 2006; Wagner *et al.*, 2006), whereas exon D is essential for Rab10 binding to myosin V tails *in vivo* (Roland *et al.*, 2009). In the present study, we found that many maize class XI myosin genes had several splicing transcripts within the randomly selected cDNA fragment in leaf and kernel (Fig. 7). Further sequence analysis showed that most of these fragments mapped to the region between the IQ motif and the tail domain, suggesting a conserved mechanism that regulates myosin function in human and plants.

*Opaque1* is the first characterized maize class XI myosin that is required for ER motility and protein body formation (Wang *et al.*, 2012a). In addition to the full-length *O1* cDNA, we isolated a short *O1* transcript that conceptually encoded a truncated, headless O1 at the O1 locus (Fig. 9). *Arabidopsis* and *Brachypodium* possess and express a headless variant of XI-K that emerged via partial myosin XI duplication (Peremyslov *et al.*, 2011). In comparison with *HDK*, the short myosin transcript is not an independent gene model but rather a headless variant of *O1* and appears to function at the transcription level. It is well known that overexpression of the myosin XI tail domain can inhibit its corresponding, endogenous, complete myosin function via the competitive binding of adaptors that mediate the interaction between myosin and its cargo. However, in the case of the headless derivative of *O1*, whether another mechanism other than dominate-negative regulation of the function of myosin exists remains to be addressed.

## Supplementary data

Supplementary data are available at *JXB* online.

Supplementary Table S1. A list of primers used in this study.

Supplementary Table S2. Sequences encoding myosin homologues retrieved from maize genome by BLASTP and TBLASTN searches.

Supplementary Table S3. Major domains presented in maize myosin proteins.

Supplementary Table S4. Analysis of intron and exon sizes and intron phases in maize myosins.

Supplementary Table S5. Analysis of repetitive sequences presented in the large introns (>1kb) in maize myosins using the RepeatMasker program.

Supplementary Fig. S1. Sequencing analysis of the alternative splicing events of some maize myosin genes.

Supplementary Fig. S2. Transcript evidence and splicing event for maize myosin genes.

Supplementary Fig. S3. Determination of the headless derivative of *O1* using two independent 5′ RACE experiments.

Supplementary Data

## Abbreviations:

ER: endoplasmic reticulum
eYFP: enhanced yellow fluorescent protein
ORF: open reading frame
RT-PCR: reverse transcription-PCR.
