# Supplementary material for: Genome-wide identification, splicing, and expression analysis of the myosin gene family in maize (Zea mays)
Source: J Exp Bot. 2013 Dec 21;65(4):923–38. doi: 10.1093/jxb/ert437 (PMC3935558; doi:10.1093/jxb/ert437)
Supplement: Supplementary Data [file supp_65_4_923__index.html]

Genome-wide identification, splicing, and expression analysis of the myosin gene family in maize (Zea mays) — Genome-wide identification, splicing, and expression analysis of the myosin gene family in maize (Zea mays) — Supplementary Data 

# Genome-wide identification, splicing, and expression analysis of the myosin gene family in maize (*Zea mays*)

## Supplementary Data

Data files

**Files in this Data Supplement:**

- Supplementary Data - Supplementary Data
