# Supplementary material for: Genome-wide identification, splicing, and expression analysis of the myosin gene family in maize (Zea mays)
Source: J Exp Bot. 2013 Dec 21;65(4):923–38. doi: 10.1093/jxb/ert437 (PMC3935558; doi:10.1093/jxb/ert437)

Genome-wide identification, splicing and expression analysis of the myosin gene family in maize (*Zea mays.*)

Guifeng Wang*****, Mingyu Zhong*****, Jiajia Wang, Jushan Zhang, Yuanping Tang, Gang Wang[§], Rentao Song[§]

Shanghai Key Laboratory of Bio-Energy Crops, School of Life Sciences, Shanghai University, No. 333 Nanchen Road, Shanghai, P.R. China

*These authors contributed equally to this work.

§Corresponding author

E-mail: wg@shu.edu.cn

E-mail: rentaosong@staff.shu.edu.cn

Tel: +86-21-66135186; fax: +86-21-66135186.

Supplementary Tables

Supplementary Table S1. A list of primers used in this study.

Usage	Primer Name	Sequence (5′--------3′)
**Semi-quantitative RT PCR**	ZmVIII-1-F	GACGGATGAGGGAGACGAA
	ZmVIII-1-R	CAGGCAGAAAGTAAAGCACAA
	ZmVIII-2-F	CAGCCTAAACCCAGACATG
	ZmVIII-2-R	CAATTCAGTAGTCCTTGACCC
	ZmVIII-3-F	CAACGACACTGGGTCAAGG
	ZmVIII-3-R	ATGCCTTGCACATTTCAACAA
	ZmXI-1-F	TGAGAAAGACAGGGAGGCA
	ZmXI-1-R	ACGCCAGAACTCCACATAACT
	ZmXI-2-F	TTTTGAGGAGGCAGGACAC
	ZmXI-2-R	ATGGATGACTGGTTGAGGC
	ZmXI-3-F	CGGTCAGCAGTTCGTTCC
	ZmXI-3-R	ACGACTCGGTAGATGTGG
	ZmXI-4-F	CAGAACGACGAGACGGGGTAG
	ZmXI-4-R	TGGACAAATTGCAGGAGAAAC
	ZmXI-5-F	CGGACATCATCAGGCTTAG
	ZmXI-5-R	TCCCGACGAAGCAGTAAG
	ZmXI-6-F	AACCGAGGAAATTGCAGAG
	ZmXI-6-R	TGACAGCGACCTACAAAGA
	ZmXI-7-F	ATGCGGGTGCTAATGACA
	ZmXI-7-R	AACTGTAAGGGCGGAATC
	ZmXI-8-F	CTCCTCTTGAGGCGTGAAT
	ZmXI-8-R	ATGTTTGGCATTGACTTCG
	ZmXI-9-F	TGACCAAGGAATCAAGCG
	ZmXI-9-R	TCTAATGCCAACCGAGCC
	ZmXI-10-F	AGGATTATTTCCACCAGCAA
	ZmXI-10-R	CTACCACCAATTAGGAAATCTA
	ZmXI-11-F	AAGTCACGACCTTTGTCCG
	ZmXI-11-R	GATTGTCGCCCAATAACCA
	ZmUBQ-Q-F	CTGGTGCCCTCTCCATATGG
	ZmUBQ-Q-R	CAACACTGACACGACTCATGACA
**Putative full length myosins gap covering**	ZmXI-2-Gap-F	ATGGGACTACAGGGTTATCAGGG
	ZmXI-2-Gap-R	GCCTCTTTGGCAGTGACATCTT
	ZmXI-6-Gap-F	TCAAAGACGGATTTCACCCTG

	ZmXI-6-Gap-R	AAGGTTCCGCAATGCCCTTT
	ZmXI-7-Gap-F	ACGGAAAGCACCAAGATG
	ZmXI-7-Gap-R	AGGCCTGAAACAAATGCA
	ZmXI-8- Gap-F1	GTGAAAGCGGTGCTGGTAAA
	ZmXI-8- Gap-R1	GCATTGTTTCCACTTGCGATA
	ZmXI-8- Gap-F2	GCCACTCTTCGTGAGATGGA
	ZmXI-8- Gap-R2	CAGTTGCCCTGGTGGCTTTA
	ZmXI-9- Gap-F	AATGGACATTGTGGGCATC
	ZmXI-9- Gap-R	CATTGCTATCGTGTTTCTGC
	ZmXI-10- Gap-F	TTGTATCAGGATTATTTCCACCAG
	ZmXI-10- Gap-R	GACTAGCCACTGCTGATCGTCT
	HDO1-5RACE1	TCATCACTACTTTTGCGAGTCACA
	HDO1-5RACE2	GCATTTCCCCTCGACTTCCTTCA
**Vector construction for subcellular localization and GUS activity assay**	O1head domain-f	GCGAATTCATGAGCTACCGGAAGGGGTTG
	O1head domain-r	CGGTCGACTCCTCCTCCTCCTCCTCCAGCACGGCGCATATCTAAAAT
	O1 head domain IQ-f	GCGAATTCATGAGCTACCGGAAGGGGTTG
	O1 head domain IQ-r	CGGTCGACTCCTCCTCCTCCTCCTCCTAGCTCCTTTCTTGCAAGCTT
	eYFP-F	GCGAATTCGTCGACATGGTGAGCAAGGGCGAGGAG
	eYFP-R	CGCTCGAGCTTGTACAGCTCGTCCATGCC
	O1-P1-F	CGTCTAGACGGGATAACCACCGTGTCAT
	O1-P1-R	ATCCCGGGCCTGCCGAAACCCTAAGCTC
	O1-P2-F	CGTCTAGACAGAGCAGGCCGCATTGATC
	O1-P2-R	ATCCCGGGGTATGGTCAGAAACCCAACGCA
**Alternatively spliced transcripts detection**	ZmXI-2-F1	AGGGTTATTTCCACAAGCA
	ZmXI-2-R1	CTCGCCCTGATAACCCTGT
	ZmXI-5-F	CGGACATCATCAGGCTTAG
	ZmXI-5-R	TCCCGACGAAGCAGTAAG
	ZmXI-7-F1	ACGGAAAGCACCAAGATG
	ZmXI-7-R1	GAATAGCCGCAACAACCC
	ZmXI-8-F	CTCCTCTTGAGGCGTGAAT
	ZmXI-8-R	ATGTTTGGCATTGACTTCG
	ZmXI-10-F	AGGATTATTTCCACCAGCAA
	ZmXI-10-R	CTACCACCAATTAGGAAATCTA
	ZmXI-11-F	AAGTCACGACCTTTGTCCG
	ZmXI-11-R	GATTGTCGCCCAATAACCA

Supplementary Table S2. Sequences encoding myosin homologs retrieved from maize genome by BLASTP and TBLASTN searches

No.	Chromosome No.	Position (bp) From	Position (bp) to	Strand	BAC Accession No.	Length (kb)	Exon	Gene Accession No.	No. of Amino Acid	MW (kDa)	pI	Domains
1	1	229190070	229205919	-	AC212453.4	15.850	23	GRMZM2G113202	1191	133.472	8.53	MH, IQ, CC
2	1	279808989	279812370	+	AC186902.4	3.382	11	GRMZM2G174083	576	65.536	9.41	MH, IQ
3	1	300108722	300149560	-	AC194061.3	40.839	38	GRMZM2G471108	1506	170.675	7.70	MH, IQ, DIL
4	2	166318980	166322361	+	AC217911.3	3.382	11	GRMZM2G066638	576	65.536	9.41	MH, IQ
5	3	203463334	203479533	+	AC186669.3	16.200	19	GRMZM2G113319	712	80.298	7.00	MH
6	3	203500963	203507842	+	AC195154.3	6.880	20	AC195154.3_FG004	1181	132.743	5.61	DIL
7	3	220883061	220902116	-	AC155377.1	19.056	39	AC155377.1_FG001	1529	173.465	8.53	MH, IQ, DIL
8	4	176870473	176897290	-	AC186563.4	26.818	38	GRMZM2G449909	1520	173.204	8.85	MH, IQ, CC, DIL
9	5	7359267	7367484	-	AC208084.3	8.218**?**	34	GRMZM2G460396	990	113.532	8.01	MH, IQ
10	5	25983026	25994059	-	AC210058.3	11.034	23	GRMZM2G139583	1194	134.072	8.50	MH, IQ, CC
11	5	36445589	36472676	+	AC211332.4	27.088	19	GRMZM2G478078	561	63.417	6.31	MH
12	5	36513549	36530714	+	AC208533.3	17.166	18	GRMZM2G110976	525	60.762	9.47	DIL
13	5	215701287	215703407	+	AC208470.3	2.121	8	GRMZM2G332687	457	50.738	6.52	MH
14	5	215703639	215714449	+	AC208470.3	10.811	30	GRMZM2G034362	1046	119.027	9.04	MH, IQ, DIL
15	6	161542870	161547726	+	AC203402.3	4.857	15	GRMZM2G476843	625	70.851	6.86	DIL
16	6	161483674	161486706	+	AC217943.4	3.033	7	GRMZM2G445423	266	29.647	7.66	MH
17	7	36208341	36213235	+	AC204070.3	4.895	12	GRMZM2G139712	457	50.738	6.52	MH
18	7	36246283	36261130	+	AC204070.3	14.848	16**?**	GRMZM2G302553	527	59.691	5.31	DIL
19	7	157571319	157580965	-	AC198603.3	9.647	24	GRMZM2G057380	1238	139.825	9.13	MH, IQ, CC
20	8	154746952	154761121	-	AC199632.4	14.170	19	GRMZM2G058155	734	83.113	7.02	MH
21	8	154701271	154704616	-	AC199632.4	3.345	12	GRMZM2G306703	493	55.523	6.44	DIL

22	9	4288567	4298608	-	AC211649.3	10.042	39	GRMZM2G435294	1529	173.418	8.92	MH, IQ, DIL

1, Grey and dark grey shading indicate the incomplete myosin sequences.
2, The paired colored sequences are two separated parts of a putative complete myosin gene.
3, Domains presented in the putative myosin sequences were determined using the PFAM and SMART databases.
4, MH, myosin head; IQ, putative calmdulin-binding motif; CC, coiled-coil; DIL, dilute.

Supplementary Table S3. Major domains presented in maize myosin proteins

No.	Accession number	Domain predicted									
		Myosin N		Head		IQ		CC		DIL	
		Range	E-value	Range	E-value	Range	E-value	Range	E-value	Range	E-value
VIII-1	GRMZM2G113202	---	---	193-849	$9e^{-225}$	867-883	0.073	981-1039	1090	---	---
						885-907	13.6				
						913-932	0.00014				
						936-953	0.025				
VIII-2	GRMZM2G139583	---	---	276-933	$2.8e^{-226}$	951-967	0.078	1065-1123	----	---	---
						969-991	1.28e				
						997-1016	0.00015				
						1020-1037	0.027				
VIII-3	GRMZM2G057380	---	---	219-875	$2.9e^{-218}$	888-910	1.32e	1023-1072	----	----	----
						911-933	1.09e				
						937-959	4.13e				
						965-982	0.0019				
XI-1	GRMZM2G471108	23-60	$2.9e^{-8}$	76-731	$2.4e^{-236}$	749-766	0.0066	888-1062	---	1325-1432	$8e^{-37}$
						711-789	0.0082				
						797-814	0.1				
						816-838	$2.74e^{2}$				
						843-862	$2.8e^{-5}$				
						867-886	0.085				
XI-2	KF493895	11-47	$6.4e^{-9}$	64-719	$7.8e^{-241}$	733-755	16	1424-1612	1610	1924-1993	$1.4e^{-19}$
						781-803	140				
						804-826	70				
						829-851	12.1				
						855-874	0.065				

XI-3	AC155377.1-FG001	11-49	$8.2e^{-13}$	64-719	$5.4e^{-242}$	736-754	0.048	---	---	1347-1453	$2.8e^{-35}$
						759-777	0.00081				
						784-802	0.0037				
						807-826	0.034				
						831-851	0.012				
						856-874	0.13				
XI-4	GRMZM2G449909	9-44	0.02	62-719	$1.8e^{-231}$	758-777	0.001	973-1054	55100	1334-1440	$3e^{-33}$
						783-802	0.0087				
						808-825	0.0004				
						831-844	0.17				
						854-874	0.031				
XI-5	GRMZM2G460396	---	---	1-653	$9e^{-238}$	670-688	0.045				
						690-712	1.9e				
						718-736	0.0023				
						738-760	$1.33e^{2}$				
						763-785	3.91e				
						789-808	0.03				
XI-6	KF493897	7-26	0.079	65-751	$3.1e^{-220}$	769-786	0.4	942-1080	2190	1335-1440	$2.3e^{-32}$
						788-810	191				
						813-835	9.73				
						836-858	61				
						864-876	0.36				
						886-906	0.3				
XI-7	KF493892	51-88	$2.5e^{-9}$	104-760	$1.1e^{-20}$	777-795	0.05	---	---	1375-1482	$8e^{-37}$
						799-819	0.0015				
						825-843	0.0065				
						848-866	0.0083				
						872-891	0.00021				
						896-915	0.00084				

XI-8	KF493894	11-48	$1.7e^{-7}$	65-610	$3.2e^{-202}$	633-652	0.0085	959-1088	2190	1794-1898	$6.9e^{-29}$
						724-742	0.69	1140-1252	1970		
						744-766	4.46	1311-1399	64400		
XI-9	KF493893	11-48	$4.8e^{-6}$	64-718	$2.1e^{-231}$	736-753	0.0011	---	---	1326-1433	$1.3e^{-35}$
						759-776	0.054				
						783-801	0.0024				
						807-825	0.12				
						830-849	$7.8e^{-6}$				
						854-873	0.0021				
XI-10	KF493896	11-46	$1.2e^{-7}$	64-719	$3.2e^{-241}$	736-754	0.9	1199-1285	1570	1698-1803	$8.7e^{-31}$
						808-825	0.95	1315-1390	1520		
						832-845	0.84				
						855-874	0.045				
XI-11	GRMZM2G435294	11-46	$9.2e^{-5}$	64-720	$3.6e^{-241}$	734-756	6.27e	---	---	1347-1453	$7.8e^{-36}$
						759-778	0.029				
						785-804	0.033				
						805-827	8.05e				
						835-852	0.029				
						856-875	0.017				

1, Domains presented in the putative myosin sequences were determined using the PFAM and SMART databases.

2, Myosin N, N-terminal SH3-like; Head, myosin head; IQ, putative calmdulin-binding motif; CC, coiled-coil; DIL, dilute.

Supplementary Table S4. Analysis of intron and exon sizes and intron phases in maize myosins

Number		Class VIII			Class XI										
		VIII-1	VIII-2	VIII-3	XI-1	XI-2	XI-3	XI-4	XI-5	XI-6	XI-7	XI-8	XI-9	XI-10	XI-11
Exon1	Size	417	564	1199	218	282	378	325	135	135	252	438	246	315	45
Intron1-2	Size	1918	1676	554	476	687	2965	957	76	158	160	770	1547	712	176
	Phase	0	0	0	0	0	0	0	-	0	0	0	0	0	0
Exon2	Size	102	102	102	144	129	129	150	144	144	144	129	129	129	129
Intron2-3	Size	85	79	80	756	2363	595	764	98	264	125	499	425	2557	110
	Phase	0	0	0	0	0	0	0	0	0	0	0	0	0	0
Exon3	Size	144	144	144	146	144	144	146	146	146	146	144	144	144	144
Intron3-4	Size	71	77	268	87	83	88	80	69	85	150	103	224	84	146
	Phase	0	0	0	2	0	0	2	2	2	2	0	0	0	0
Exon4	Size	151	151	151	157	146	146	160	157	157	157	146	146	146	146
Intron4-5	Size	79	76	79	319	103	108	378	101	108	95	113	79	104	214
	Phase	1	1	1	0	2	2	0	0	0	0	2	2	2	2
Exon5	Size	28	28	28	59	157	157	59	59	59	59	157	157	157	157
Intron5-6	Size	844	836	90	93	137	114	304	69	318	96	148	203	138	742
	Phase	2	2	2	2	0	0	2	2	2	2	0	0	0	0
Exon6	Size	166	166	166	160	59	59	157	160	157	160	59	59	59	59
Intron6-7	Size	84	75	88	447	6372	91	1353	88	1439	201	167	128	4646	121
	Phase	0	0	0	0	2	2	0	0	0	0	2	2	2	2
Exon7	Size	64	64	64	150	160	160	150	150	150	150	160	160	160	160
Intron7-8	Size	107	108	113	84	98	111	134	109	100	89	72	184	98	84
	Phase	1	1	1	0	0	0	0	0	0	0	0	0	0	0
Exon8	Size	14	14	14	137	150	150	137	137	137	137	150	150	150	150
Intron8-9	Size	91	85	80	86	92	81	491	79	2379	86	84	85	107	98
	Phase	0	0	0	2	0	0	2	2	2	2	0	0	0	0

**Exon9**	**Size**	85	85	85	147	137	137	147	147	147	147	137	137	137	137
**Intron9-10**	**Size**	211	213	187	111	95	90	70	119	71	127	149	78	100	92
	**Phase**	1	1	1	2	2	2	2	2	2	2	2	2	2	2
**Exon10**	**Size**	104	104	104	102	147	147	102	102	102	102	147	147	147	147
**Exon10-11**	**Size**	111	109	91	3641	74	89	360	90	868	670	82	99	74	100
	**Phase**	0	0	0	2	2	2	2	2	2	2	2	2	2	2
**Exon11**	**Size**	139	139	139	58	102	102	58	58	58	58	102	102	102	102
**Intron11-12**	**Size**	776	795	106	114	96	89	85	104	81	100	93	4282	95	81
	**Phase**	1	1	1	0	2	2	0	0	0	0	2	2	2	2
**Exon12**	**Size**	119	119	119	102	58	58	102	102	102	102	58	58	58	58
**Intron12-13**	**Size**	5068	97	84	943	83	75	1140	80	17674	402	78	82	83	101
	**Phase**	0	0	0	0	0	0	0	0	0	0	0	0	0	0
**Exon13**	**Size**	153	153	153	38	102	102	38	38	38	38	102	102	102	102
**Intron13-14**	**Size**	79	251	79	90	199	1304	84	134	74	109	111	331	1387	661
	**Phase**	0	0	0	2	0	0	2	2	2	2	0	0	0	0
**Exon14**	**Size**	90	90	90	127	38	35	127	127	127	127	38	38	38	38
**Intron14-15**	**Size**	379	341	882	3111	291	144	89	84	98	76	149	70	105	91
	**Phase**	0	0	0	0	2	2	0	0	0	0	2	2	2	2
**Exon15**	**Size**	78	78	78	168	127	127	174	171	174	171	127	127	127	127
**Intron15-16**	**Size**	1167	1143	583	73	121	88	549	72	429	81	85	8890	118	109
	**Phase**	0	0	0	0	0	0	0	0	0	0	0	0	0	0
**Exon16**	**Size**	159	159	159	132	168	171	132	132	132	132	165	165	168	171
**Intron16-17**	**Size**	112	110	148	755	2618	4508	1384	111	169	82	24525	76	1079	100
	**Phase**	0	0	0	0	0	0	0	0	0	0	0	0	0	0
**Exon17**	**Size**	204	204	201	110	132	132	110	107	110	110	277	132	132	132
**Intron17-18**	**Size**	90	90	363	75	84	70	586	128	148	87	105	847	84	85
	**Phase**	0	0	0	2	0	0	2	2	2	2	1	0	0	0

**Exon18**	**Size**	206	206	206	61	110	110	61	61	90	61	89	110	110s	110
**Intron18-19**	**Size**	72	79	95	285	80	93	6631	114	243	1676	123	82	82	111
	**Phase**	2	2	2	0	2	2	0	0	2	0	0	2	2	2
**Exon19**	**Size**	133	136	136	178	61	61	178	178	61	178	120	61	61	61
**Intron19-20**	**Size**	191	190	153	103	9463	149	1023	100	22499	104	89	583	?	77
	**Phase**	0	0	0	1	0	0	1	1	0	1	0	0	0	0
**Exon20**	**Size**	130	139	130	206	178	178	206	206	178	206	99	178	178	178
**Intron20-21**	**Size**	105	102	136	378	97	148	180	78	18061	598	85	90	?	76
	**Phase**	1	1	1	0	1	1	0	0	1	0	0	1	1	1
**Exon21**	**Size**	147	147	147	120	206	206	120	120	206	120	189	206	206	206
**Intron21-22**	**Size**	99	100	86	257	145	117	210	83	222	117	237	2436	?	132
	**Phase**	1	1	1	0	0	0	0	0	0	0	0	0	0	0
**Exon22**	**Size**	80	80	125	99	120	120	99	99	120	99	140	120	120	120
**Intron22-23**	**Size**	145	139	316	14936	74	89	609	90	2392	652	10185	4129	?	98
	**Phase**	0	0	0	0	0	0	0	0	0	0	2	0	0	0
**Exon23**	**Size**	1053	931	207	210	99	99	222	216	99	213	168	99	99	99
**Intron23-24**	**Size**			83	100	167	94	160	84	266	139	3460	?	?	161
	**Phase**			0	0	0	0	0	0	0	0	2	0	0	0
**Exon24**	**Size**			956	140	189	222	140	140	222	140	168	210	189	219
**Intron24-25**	**Size**				81	229	307	119	87	292	128	4038	?	?	167
	**Phase**				2	0	0	2	2	0	2	2	0	0	0
**Exon25**	**Size**				109	140	140	112	53	119	121	168	140	140	140
**Intron25-26**	**Size**				210	2251	258	268	348	73	88	137	99	?	110
	**Phase**				0	2	2	0	1	2	0	2	2	2	2
**Exon26**	**Size**				36	168	115	51	167	91	39	167	109	14	112
**Intron26-27**	**Size**				75	219	91	91	109	244	89	92	159	5798	97
	**Phase**				0	2	0	0	0	0	0	1	0	1	0

**Exon27**	**Size**	57	168	54	90	101	84	84	148	39	148	51
**Intron27-28**	**Size**	89	176	97	259	188	136	71	93	78	15389	101
	**Phase**	0	2	0	0	2	0	0	2	0	2	0
**Exon28**	**Size**	171	168	81	171	181	171	171	168	78	168	81
**Intron28-29**	**Size**	120	150	126	95	106	76	562	111	99	164	85
	**Phase**	0	2	0	0	0	0	0	2	0	2	0
**Exon29**	**Size**	150	168	171	144	156	150	156	168	171	168	171
**Intron29-30**	**Size**	4893	76	699	1772	312	867	79	7925	234	100	108
	**Phase**	0	2	0	0	0	0	0	2	0	2	0
**Exon30**	**Size**	204	168	153	177	207	177	186	136	150	168	156
**Intron30-31**	**Size**	1280	77	93	76	74	140	137	809	564	239	86
	**Phase**	0	2	0	0	0	0	0	0	0	2	0
**Exon31**	**Size**	105	168	207	138	147	138	135	161	174	168	207
**Intron31-32**	**Size**	83	1301	82	126	85	1042	81	96	1826	91	83
	**Phase**	0	2	0	0	0	0	0	2	0	2	0
**Exon32**	**Size**	71	168	141	71	71	71	71	210	153	210	144
**Intron32-33**	**Size**	77	77	86	321	137	87	78	111	73	107	152
	**Phase**	2	2	0	2	2	2	2	2	0	2	0
**Exon33**	**Size**	100	168	71	100	100	97	100	124	71	126	71
**Intron33-34**	**Size**	480	5244	74	541	216	81	70	76	90	83	100
	**Phase**	0	2	2	0	0	0	0	0	2	2	2
**Exon34**	**Size**	57	168	100	57	121	57	57	93	100	91	100
**Intron34-35**	**Size**	250	155	76	86		8665	196	118	586	10073	117
	**Phase**	0	2	0	0		0	0	0	0	0	0
**Exon35**	**Size**	57	189	57	57		57	57	72	57	81	57
**Intron35-36**	**Size**	75	58	80	90		219	128	89	8200	75	99
	**Phase**	0	2	0	0		0	0	0	0	0	0

**Exon36**	**Size**		81	168	57	81		81	81	186	57	195	57
**Intron36-37**	**Size**		73	579	94	89		75	262	157	297	344	86
	**Phase**		0	2	0	0		0	0	0	0	0	0
**Exon37**	**Size**		89	168	81	83		83	83	345	81	339	81
**Intron37-38**	**Size**		105	423	118	133		119	89	79	337	79	79
	**Phase**		2	2	0	2		2	2	0	0	0	0
**Exon38**	**Size**		706	168	83	499		444	20	138	86	132	83
**Intron38-39**	**Size**			105	85				130	82	79	91	97
	**Phase**			2	2				1	0	2	0	2
**Exon39**	**Size**			165	549				95	71	627	71	201
**Intron39-40**	**Size**			134						92		73	
	**Phase**			2						2		2	
**Exon40**	**Size**			168						100		100	
**Intron40-41**	**Size**			189						1231		73	
	**Phase**			2						0		0	
**Exon41**	**Size**			210						57		57	
**Intron41-42**	**Size**			111						110		235	
	**Phase**			2						0		0	
**Exon42**	**Size**			121						57		57	
**Intron42-43**	**Size**			80						405		82	
	**Phase**			0						0		0	
**Exon43**	**Size**			93						81		81	
**Intron43-44**	**Size**			94						89		100	
	**Phase**			0						0		0	
**Exon44**	**Size**			81						83		83	
**Intron44-45**	**Size**			77						101		99	
	**Phase**			0						2		2	

**Exon45**	**Size**		195			115	575
**Intron45-46**	**Size**		348				
	**Phase**		0				
**Exon46**	**Size**		345				
**Intron46-47**	**Size**		79				
	**Phase**		0				
**Exon47**	**Size**		132				
**Intron47-48**	**Size**		86				
	**Phase**		0				
**Exon48**	**Size**		71				
**Intron48-49**	**Size**		79				
	**Phase**		2				
**Exon49**	**Size**		100				
**Intron49-50**	**Size**		75				
	**Phase**		0				
**Exon50**	**Size**		57				
**Intron50-51**	**Size**		130				
	**Phase**		0				
**Exon51**	**Size**		480				
**Intron51-52**	**Size**		80				
	**Phase**		0				
**Exon52**	**Size**		441				

The number colored in red represents the uncertain fragment length because of the loss of the genomic data in current maize genome release.

Supplementary Table S5. Analysis of repetitive sequences presented in the large introns (> 1 kb) in maize myosins using the RepeatMasker program

No. of Genes	No. of Intron	Retroelements			DNA Transposon			Others		
		Length	Percentage	Clade	Length	Percentage	Clade	Length	Percentage	Clade
**VIII-1**	1-2	-	-	-	296	15.43	-	51	2.66	Low complexity
	12-13	4203	82.93	Ty1/Copia	-	-	-	-	-	-
	15-16	-	-	-	-	-	-	-	-	-
**VIII-2**	1-2	-	-	-	408(2)<br>233<br>178	24.34<br>13.90<br>10.62	hobo-Activator<br>Tc1-IS630-Pogo<br>Tourist/Harbinger	-	-	-
	15-16	-	-	-	-	-	-	-	-	-
**VIII-3**	-	-	-	-	-	-	-	-	-	-
**XI-1**	10-11	1518(2)<br>266	41.69<br>7.31	L1/CIN4<br>Ty1/Copia	299(2)	8.21	Tourist/Harbinger	-	-	-
	14-15	1068<br>899	34.33<br>28.90	L1/CIN4<br>Ty1/Copia	155	4.98	hobo-Activator	-	-	-
	22-23	146<br>6883(5)<br>5257(5)	0.98<br>46.08<br>35.20	SINEs<br>L1/CIN4<br>Ty1/Copia	-	-	-	146<br>26	0.98<br>0.17	Small RNA<br>Simple repeats
	29-30	1050<br>2569	21.46<br>52.50	L1/CIN4<br>Ty1/Copia	165	3.37	hobo-Activator	-	-	-
	30-31	125	9.77	L1/CIN4	-	-	-	-	-	-
**XI-2**	2-3	514	21.75	L1/CIN4	-	-	-	-	-	-

		497(2)	21.03	Ty1/Copia						
	6-7	3593(3)	62.04	Ty1/Copia	655	10.28	hobo-Activator	-	-	-
	16-17	57<br>1193	2.18<br>45.57	L1/CIN4<br>Ty1/Copia	-	-	-	-	-	-
	19-20	6928(4)	73.21	L1/CIN4	88	0.93	hobo-Activator	-	-	-
	25-26	1223<br>215	54.33<br>9.55	L1/CIN4<br>Gypsy/DIRS1						
	29-30	3250(2)	40.54	Ty1/Copia	148(2)	1.85	Tourist/Harbinger	149<br>133(4)	1.86<br>1.66	Unclassified<br>Simple repeats
**XI-3**	1-2	284	9.58	L1/CIN4	212	7.15	-	-	-	-
	13-14	-	-	-	-	-	-	27	2.07	Simple repeats
	16-17	371<br>1892(3)	8.23<br>41.97	L1/CIN4<br>Gypsy/DIRS1	-	-	-	-	-	-
**XI-4**	6-7	316	23.36	RTE/Bov-B	-	-	-	-	-	-
	12-13	-	-	-	-	-	-	-	-	-
	16-17	-	-	-	84	6.07	Tc1-IS630-Pogo	-	-	-
	18-19	105<br>4279	1.58<br>64.53	L1/CIN4<br>Ty1/Copia	130	1.96	Tourist/Harbinger	-	-	-
	19-20	327	31.96	RTE/Bov-B	-	-	-	-	-	-

	29-30	767(3)<br>118	43.28<br>6.66	L1/CIN4<br>Ty1/Copia	146	8.24	hobo-Activator	-	-	-
**XI-5**	-	-	-	-	-	-	-	-	-	-
**XI-6**	6-7	415	28.84	L1/CIN4	-	-	-	51	3.54	Unclassified
	8-9	1713	72.01	Ty1/Copia	-	-	-	-	-	-
	12-13	2007(2)<br>7550(3)<br>5170(3)	11.36<br>42.72<br>29.25	RTE/Bov-B<br>L1/CIN4<br>Ty1/Copia	-	-	-	-	-	-
	19-20	46<br>7617(9)<br>4512(6)<br>4167	0.2<br>33.85<br>20.05<br>18.52	RTE/Bov-B<br>L1/CIN4<br>Ty1/Copia<br>Gypsy/DIRS1	-	-	-	23	0.1	Simple repeats
	20-21	13171(2)<br>930	72.93<br>5.15	Ty1/Copia<br>Gypsy/DIRS1	-	-	-	-	-	-
	22-23	502<br>54	20.99<br>2.26	RTE/Bov-B<br>L1/CIN4	127	5.31	Tourist/Harbinger	-	-	-
	31-32	-	-	-	228(2)	21.88	Tourist/Harbinger	-	-	-
	34-35	5443(2)<br>609(2)	62.82<br>7.03	L1/CIN4<br>Ty1/Copia	123	1.42	Tc1-IS630-Pogo	-	-	-
**XI-7**	18-19	61<br>223	3.64<br>13.31	RTE/Bov-B<br>L1/CIN4	166	9.90	Tc1-IS630-Pogo	-	-	-
**XI-8**	16-17	2620(3)<br>13201(9)<br>892(3)	10.68<br>53.83<br>3.64	L1/CIN4<br>Ty1/Copia<br>Gypsy/DIRS1	138	0.56	hobo-Activator	110<br>157(2)	0.45<br>0.64	Unclassified<br>Simple repeats

	22-23	7451(3)	73.16	Gypsy/DIRS1	863(3)<br>83	8.47<br>0.42	Tourist/Harbinger<br>-	-	-	-
	23-24	1309(6)<br>56<br>149	37.83<br>1.62<br>4.31	L1/CIN4<br>Gypsy/DIRS1<br>SINEs	-	-	-	149	4.31	Small RNA
	24-25	2300(4)<br>132<br>36	56.96<br>3.27<br>0.89	L1/CIN4<br>Ty1/Copia<br>Gypsy/DIRS1	291	7.21	Tourist/Harbinger	-	-	-
	29-30	2644<br>3670(5)<br>301	33.36<br>46.31<br>3.80	L1/CIN4<br>Ty1/Copia<br>-	-	-	-	32	0.40	Simple repeats
	40-41	114<br>328	9.26<br>26.65	L1/CIN4<br>Gypsy/DIRS1	-	-	-	-	-	-
**XI-9**	1-2	-	-	-	382	24.69	hobo-Activator	-	-	-
	11-12	531(2)<br>190(2)	12.40<br>4.44	L1/CIN4<br>Gypsy/DIRS1	-	-	-	-	-	-
	15-16	1474<br>3283(3)	16.58<br>36.93	L1/CIN4<br>Ty1/Copia	285(2)	3.21	hobo-Activator	62<br>91	0.70<br>1.02	Unclassified<br>Simple repeats
	21-22	1888	77.50	RTE/Bov-B	-	-	-	-	-	-
	22-23	1477	35.77	Ty1/Copia	-	-	-	-	-	-
	23-24	189(2)<br>4980(2)<br>1917	1.88<br>49.54<br>19.07	L1/CIN4<br>Ty1/Copia<br>Gypsy/DIRS1	863	8.58	hobo-Activator	87(3)	0.87	Simple repeats

	30-31	125(2)<br>123	6.85<br>6.74	L1/CIN4<br>Ty1/Copia	-	-	-	-	-	-
	35-36	1052(2)<br>963(2)<br>2095	12.83<br>11.74<br>20.55	L1/CIN4<br>Ty1/Copia<br>Gypsy/DIRS1	278(2)	3.39	Tourist/Harbinger	36	0.44	Low complexity
**XI-10**	2-3	-	-	-	-	-	-	-	-	-
	6-7	2532(3)<br>609(3)	54.50<br>13.11	L1/CIN4<br>Ty1/Copia	35	0.75	hobo-Activator	-	-	-
	13-14	688<br>60	49.60<br>4.33	RTE/Bov-B<br>L1/CIN4	-	-	-	51<br>47	3.68<br>3.39	Unclassified<br>Simple repeats
	16-17	197(2)	18.26	L1/CIN4	-	-	-	50	4.63	Unclassified
	19-20	3786(6)<br>3516(4)	40.15<br>37.29	L1/CIN4<br>Ty1/Copia	-	-	-	-	-	-
	26-27	1778(2)	30.67	L1/CIN4	70	1.21	Tourist/Harbinger	-	-	-
	27-28	9256(6)<br>46	60.15<br>0.30	Ty1/Copia<br>Gypsy/DIRS1	-	-	-	193(3)	1.25	Simple repeats
	34-35	8063(7)<br>398(2)	80.5<br>3.95	L1/CIN4<br>Gypsy/DIRS1	-	-	-	27	0.27	Simple repeats
**XI-11**	-	-	-	-	-	-	-	-	-	-

Supplementary Figures

Supplementary Figure S1. Sequencing analysis of the alternative splicing events of some maize myosin genes.

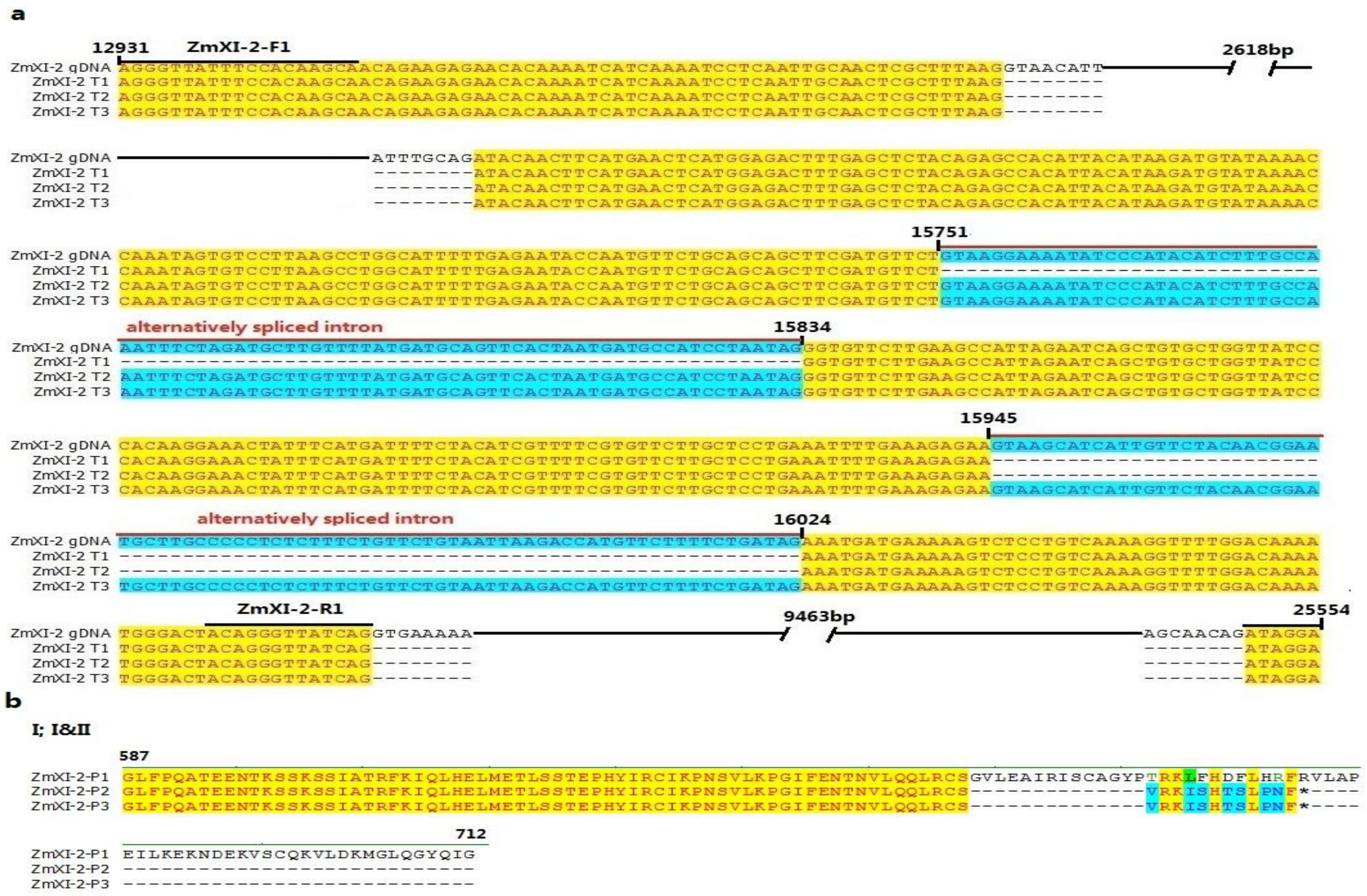

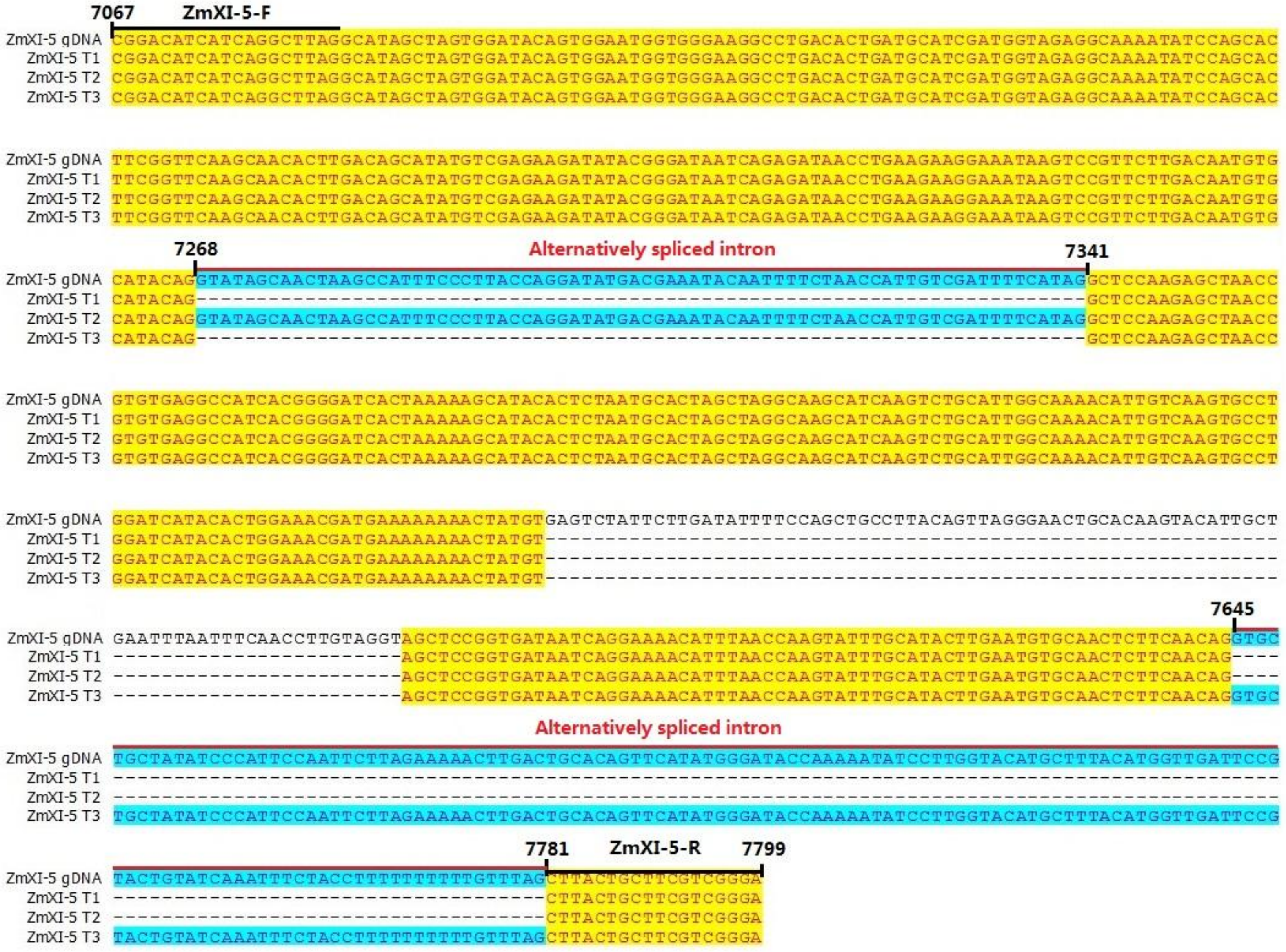

7067
ZmXI-5-F
ZmXI-5 gDNA
ZmXI-5 T1
ZmXI-5 T2
ZmXI-5 T3
7268
Alternatively spliced intron
7341
7645
Alternatively spliced intron
7781
ZmXI-5-R
7799

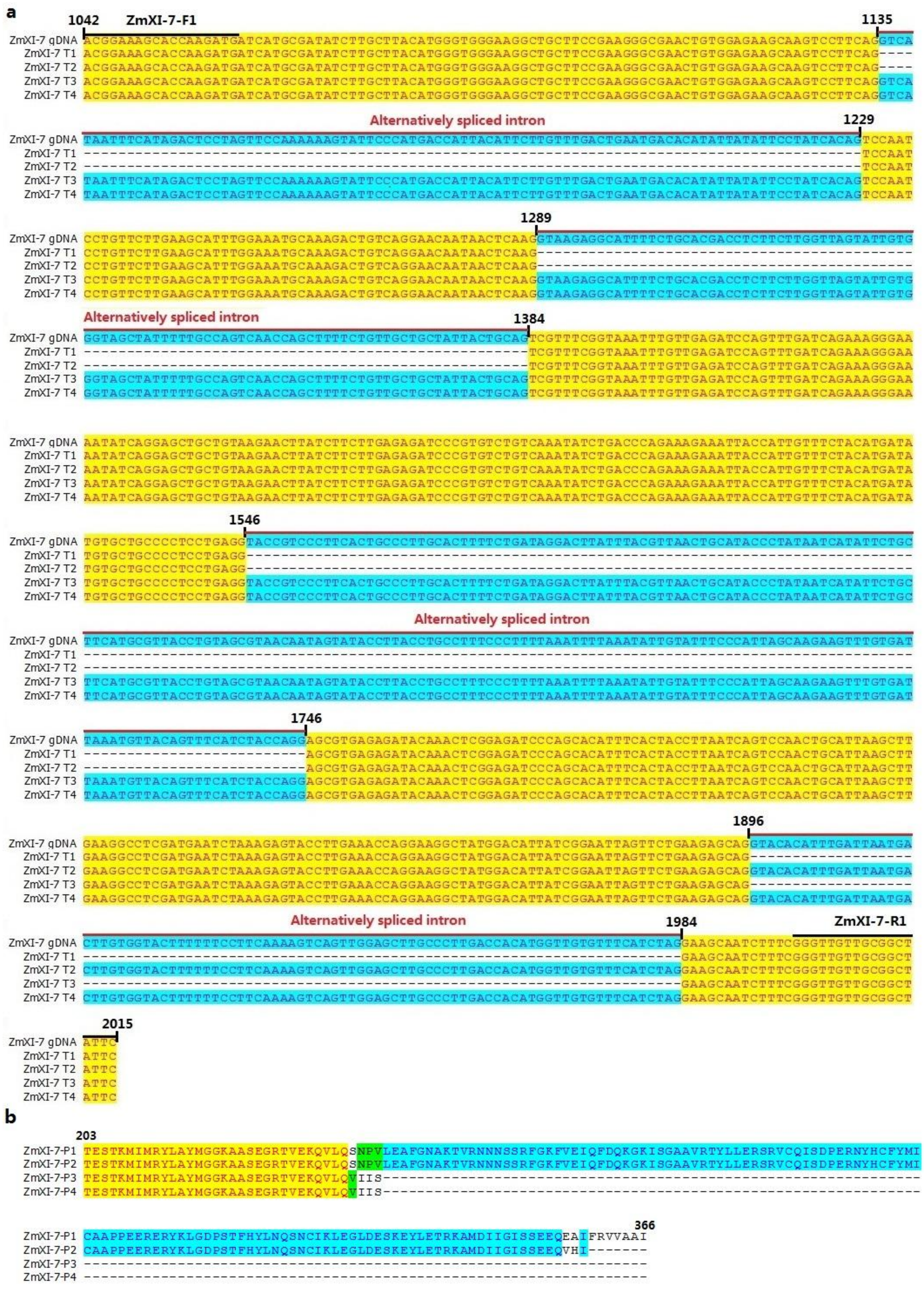
a
1042
ZmXI-7-F1
1135
1229
1289
1384
1546
1746
1896
1984
ZmXI-7-R1
2015
Alternatively spliced intron
Alternatively spliced intron
Alternatively spliced intron
Alternatively spliced intron
ZmXI-7 gDNA
ZmXI-7 T1
ZmXI-7 T2
ZmXI-7 T3
ZmXI-7 T4
b
203
366
ZmXI-7-P1
ZmXI-7-P2
ZmXI-7-P3
ZmXI-7-P4

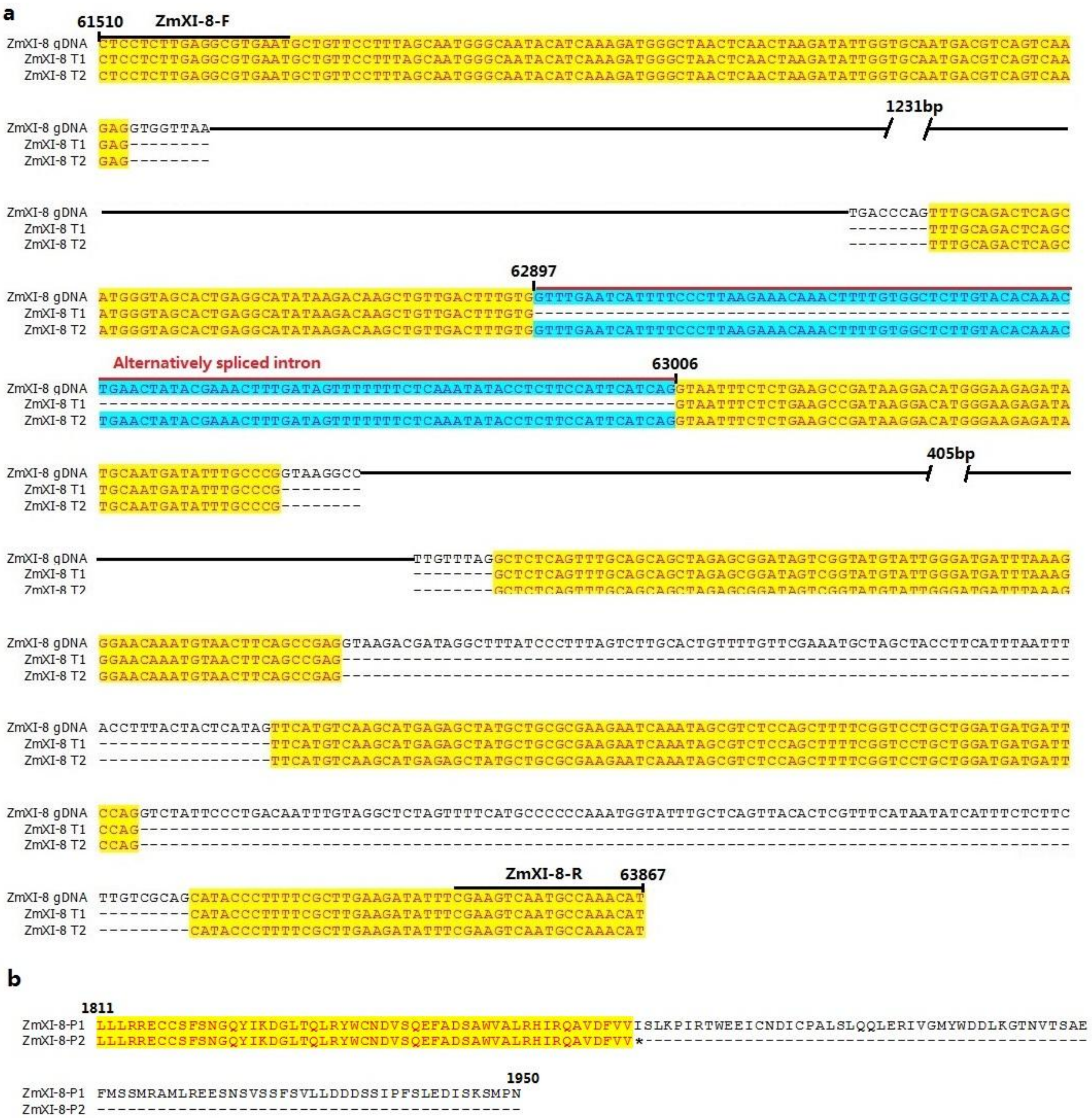
a
61510
ZmXI-8-F
ZmXI-8 gDNA CTCCTCTTGAGGCGTGAATGCTGTTCCTTTAGCAATGGGCAATACATCAAAGATGGGCTAACTCAACTAAGATATTGGTGCAATGACGTCAGTCAA
ZmXI-8 T1 CTCCTCTTGAGGCGTGAATGCTGTTCCTTTAGCAATGGGCAATACATCAAAGATGGGCTAACTCAACTAAGATATTGGTGCAATGACGTCAGTCAA
ZmXI-8 T2 CTCCTCTTGAGGCGTGAATGCTGTTCCTTTAGCAATGGGCAATACATCAAAGATGGGCTAACTCAACTAAGATATTGGTGCAATGACGTCAGTCAA
1231bp
ZmXI-8 gDNA GAGGTGGTTAA
ZmXI-8 T1 GAG--------
ZmXI-8 T2 GAG--------
ZmXI-8 gDNA TGACCCAGTTTGCAGACTCAGC
ZmXI-8 T1 --------TTTGCAGACTCAGC
ZmXI-8 T2 --------TTTGCAGACTCAGC
62897
ZmXI-8 gDNA ATGGGTAGCACTGAGGCATATAAGACAAGCTGTTGACTTTGTGGTTTGAATCATTTTCCCTTAAGAAACAAACTTTTGTGGCTCTTGTACACAAAC
ZmXI-8 T1 ATGGGTAGCACTGAGGCATATAAGACAAGCTGTTGACTTTGTG--
ZmXI-8 T2 ATGGGTAGCACTGAGGCATATAAGACAAGCTGTTGACTTTGTGGTTTGAATCATTTTCCCTTAAGAAACAAACTTTTGTGGCTCTTGTACACAAAC
Alternatively spliced intron
63006
ZmXI-8 gDNA TGAACTATACGAAACTTTGATAGTTTTTTTCTCAAATATACCTCTTCCATTCATCAGGTAATTTCTCTGAAGCCGATAAGGACATGGGAAGAGATA
ZmXI-8 T1 --GTAATTTCTCTGAAGCCGATAAGGACATGGGAAGAGATA
ZmXI-8 T2 TGAACTATACGAAACTTTGATAGTTTTTTTCTCAAATATACCTCTTCCATTCATCAGGTAATTTCTCTGAAGCCGATAAGGACATGGGAAGAGATA
405bp
ZmXI-8 gDNA TGCAATGATATTTGCCCGGTAAGGCC
ZmXI-8 T1 TGCAATGATATTTGCCCG--------
ZmXI-8 T2 TGCAATGATATTTGCCCG--------
ZmXI-8 gDNA TTGTTTAGGCTCTCAGTTTGCAGCAGCTAGAGCGGATAGTCGGTATGTATTGGGATGATTTAAAG
ZmXI-8 T1 --------GCTCTCAGTTTGCAGCAGCTAGAGCGGATAGTCGGTATGTATTGGGATGATTTAAAG
ZmXI-8 T2 --------GCTCTCAGTTTGCAGCAGCTAGAGCGGATAGTCGGTATGTATTGGGATGATTTAAAG
ZmXI-8 gDNA GGAACAAATGTAACTTCAGCCGAGGTAAGACGATAGGCTTTATCCCTTTAGTCTTGCACTGTTTTGTTCGAAATGCTAGCTACCTTCATTTAATTT
ZmXI-8 T1 GGAACAAATGTAACTTCAGCCGAG---
ZmXI-8 T2 GGAACAAATGTAACTTCAGCCGAG---
ZmXI-8 gDNA ACCTTTACTACTCATAGTTCATGTCAAGCATGAGAGCTATGCTGCGCGAAGAATCAAATAGCGTCTCCAGCTTTTCGGTCCTGCTGGATGATGATT
ZmXI-8 T1 -----------------TTCATGTCAAGCATGAGAGCTATGCTGCGCGAAGAATCAAATAGCGTCTCCAGCTTTTCGGTCCTGCTGGATGATGATT
ZmXI-8 T2 -----------------TTCATGTCAAGCATGAGAGCTATGCTGCGCGAAGAATCAAATAGCGTCTCCAGCTTTTCGGTCCTGCTGGATGATGATT
ZmXI-8 gDNA CCAGGTCTATTCCCTGACAATTTGTAGGCTCTAGTTTTCATGCCCCCCAAATGGTATTTGCTCAGTTACACTCGTTTCATAATATCATTTCTCTTC
ZmXI-8 T1 CCAG---
ZmXI-8 T2 CCAG---
ZmXI-8-R
63867
ZmXI-8 gDNA TTGTCGCAGCATACCCTTTTCGCTTGAAGATATTTCGAAGTCAATGCCAAACAT
ZmXI-8 T1 ---------CATACCCTTTTCGCTTGAAGATATTTCGAAGTCAATGCCAAACAT
ZmXI-8 T2 ---------CATACCCTTTTCGCTTGAAGATATTTCGAAGTCAATGCCAAACAT
b
1811
ZmXI-8-P1 LLLRRECCSFSNGQYIKDGLTQLRYWCNDVSQEFADSAWVALRHIRQAVDFVVISLKPIRTWEEICNDICPALSLQQLERIVGMYWDDLKGTNVTSAE
ZmXI-8-P2 LLLRRECCSFSNGQYIKDGLTQLRYWCNDVSQEFADSAWVALRHIRQAVDFVV*---
1950
ZmXI-8-P1 FMSSMRAMLREESNSVSSFSVLLDDDSSIPFSLEDISKSMPN
ZmXI-8-P2 ---

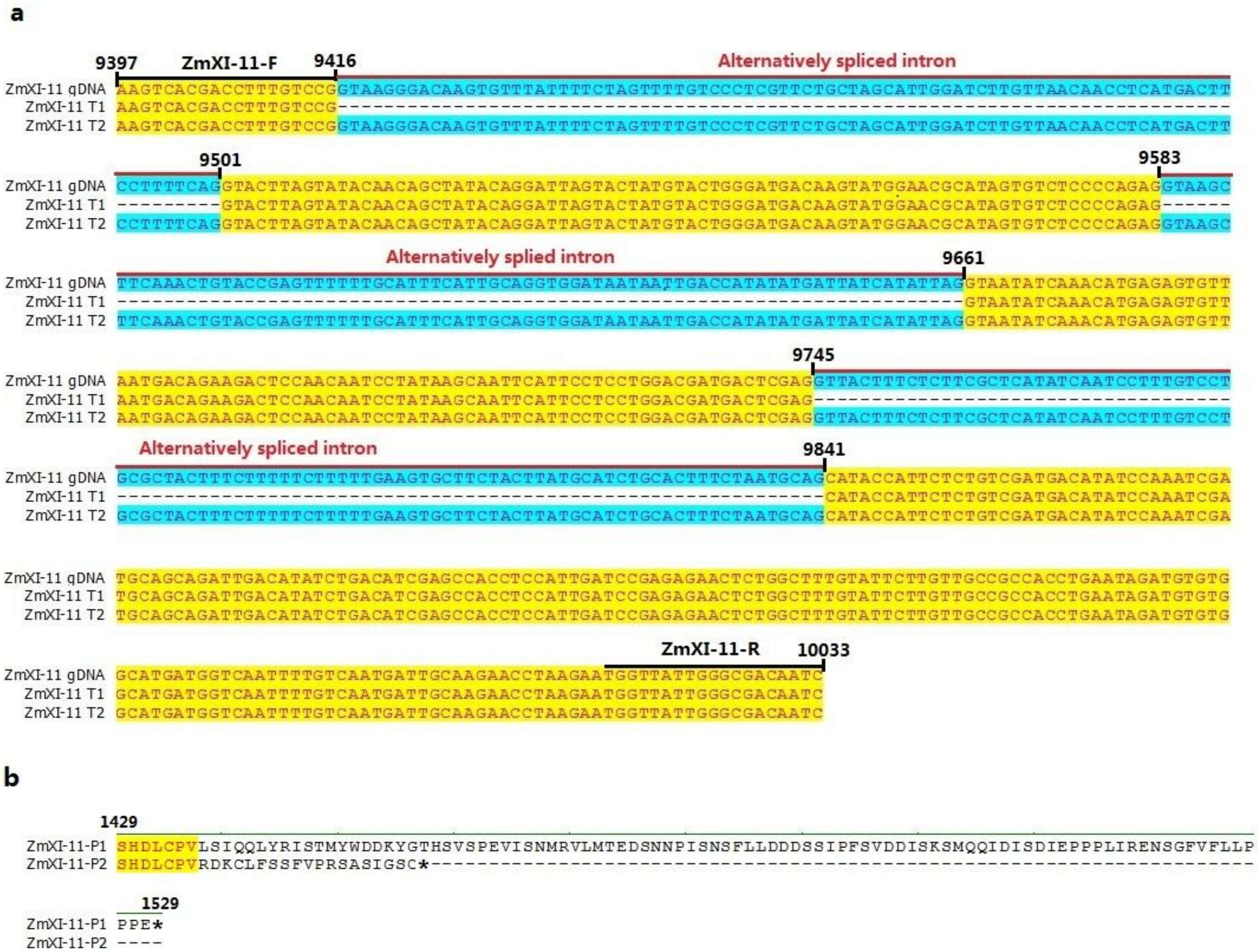
a
9397
ZmXI-11-F
9416
Alternatively spliced intron
ZmXI-11 gDNA AAGTCACGACCTTTGTCCGGTAAGGGACAAGTGTTTATTTTCTAGTTTTGTCCCTCGTTCTGCTAGCATTGGATCTTGTTAACAACCTCATGACTT
ZmXI-11 T1 AAGTCACGACCTTTGTCCG---
ZmXI-11 T2 AAGTCACGACCTTTGTCCGGTAAGGGACAAGTGTTTATTTTCTAGTTTTGTCCCTCGTTCTGCTAGCATTGGATCTTGTTAACAACCTCATGACTT
9501
9583
ZmXI-11 gDNA CCTTTTCAGGTACTTAGTATACAACAGCTATACAGGATTAGTACTATGTACTGGGATGACAAGTATGGAACGCATAGTGTCTCCCCAGAGGTAAGC
ZmXI-11 T1 ---------GTACTTAGTATACAACAGCTATACAGGATTAGTACTATGTACTGGGATGACAAGTATGGAACGCATAGTGTCTCCCCAGAG------
ZmXI-11 T2 CCTTTTCAGGTACTTAGTATACAACAGCTATACAGGATTAGTACTATGTACTGGGATGACAAGTATGGAACGCATAGTGTCTCCCCAGAGGTAAGC
Alternatively splied intron
9661
ZmXI-11 gDNA TTCAAACTGTACCGAGTTTTTTGCATTTCATTGCAGGTGGATAATAATTGACCATATATGATTATCATATTAGGTAATATCAAACATGAGAGTGTT
ZmXI-11 T1 ---GTAATATCAAACATGAGAGTGTT
ZmXI-11 T2 TTCAAACTGTACCGAGTTTTTTGCATTTCATTGCAGGTGGATAATAATTGACCATATATGATTATCATATTAGGTAATATCAAACATGAGAGTGTT
9745
ZmXI-11 gDNA AATGACAGAAGACTCCAACAATCCTATAAGCAATTCATTCCTCCTGGACGATGACTCGAGGTTACTTTCTCTTCGCTCATATCAATCCTTTGTCCT
ZmXI-11 T1 AATGACAGAAGACTCCAACAATCCTATAAGCAATTCATTCCTCCTGGACGATGACTCGAG------------------------------------
ZmXI-11 T2 AATGACAGAAGACTCCAACAATCCTATAAGCAATTCATTCCTCCTGGACGATGACTCGAGGTTACTTTCTCTTCGCTCATATCAATCCTTTGTCCT
Alternatively spliced intron
9841
ZmXI-11 gDNA GCGCTACTTTCTTTTTCTTTTTGAAGTGCTTCTACTTATGCATCTGCACTTTCTAATGCAGCATACCATTCTCTGTCGATGACATATCCAAATCGA
ZmXI-11 T1 ---CATACCATTCTCTGTCGATGACATATCCAAATCGA
ZmXI-11 T2 GCGCTACTTTCTTTTTCTTTTTGAAGTGCTTCTACTTATGCATCTGCACTTTCTAATGCAGCATACCATTCTCTGTCGATGACATATCCAAATCGA
ZmXI-11 gDNA TGCAGCAGATTGACATATCTGACATCGAGCCACCTCCATTGATCCGAGAGAACTCTGGCTTTGTATTCTTGTTGCCGCCACCTGAATAGATGTGTG
ZmXI-11 T1 TGCAGCAGATTGACATATCTGACATCGAGCCACCTCCATTGATCCGAGAGAACTCTGGCTTTGTATTCTTGTTGCCGCCACCTGAATAGATGTGTG
ZmXI-11 T2 TGCAGCAGATTGACATATCTGACATCGAGCCACCTCCATTGATCCGAGAGAACTCTGGCTTTGTATTCTTGTTGCCGCCACCTGAATAGATGTGTG
ZmXI-11-R
10033
ZmXI-11 gDNA GCATGATGGTCAATTTTGTCAATGATTGCAAGAACCTAAGAATGGTTATTGGGCGACAATC
ZmXI-11 T1 GCATGATGGTCAATTTTGTCAATGATTGCAAGAACCTAAGAATGGTTATTGGGCGACAATC
ZmXI-11 T2 GCATGATGGTCAATTTTGTCAATGATTGCAAGAACCTAAGAATGGTTATTGGGCGACAATC
b
1429
ZmXI-11-P1 SHDLCPVLSIQQLYRISTMYWDDKYGTHSVSPEVISNMRVLMTEDSNNPISNSFLLDDDSSIPFSVDDISKSMQQIDISDIEPPPLIRENSGFVFLLP
ZmXI-11-P2 SHDLCPVRDKCLFSSFVPRSASIGSC*
1529
ZmXI-11-P1 PPE*
ZmXI-11-P2 ----

Supplementary Figure S2. Transcript evidence and splicing event for maize myosin genes.

ZmVIII-1

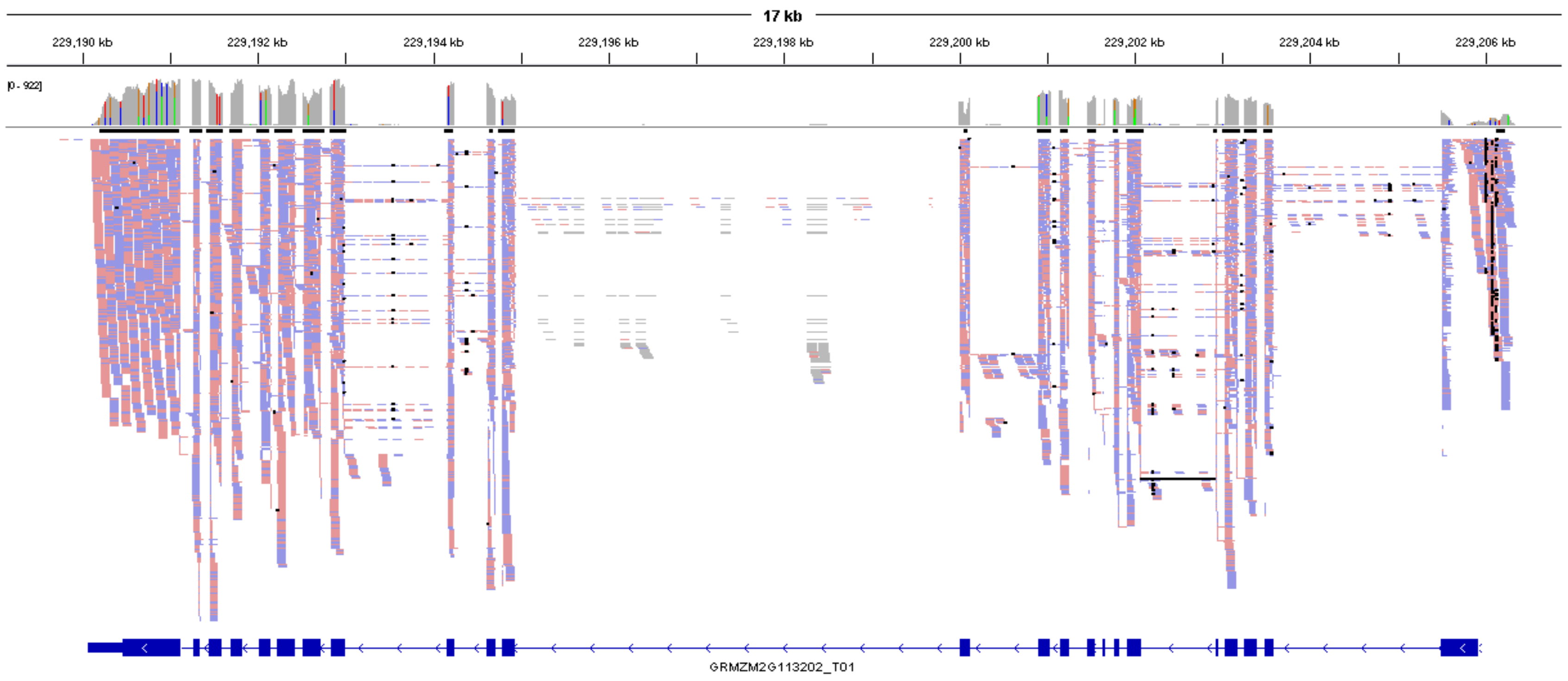

ZmVIII-2

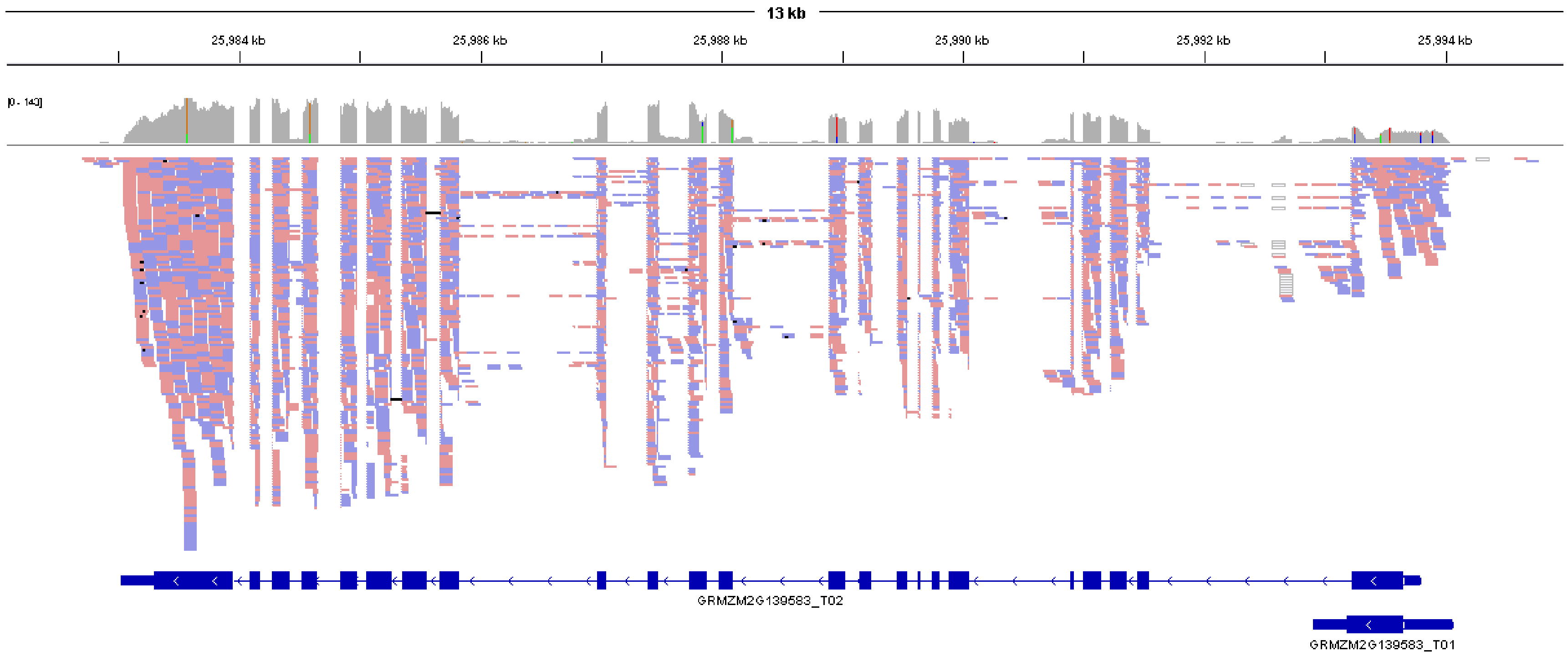

13 kb
25,984 kb
25,986 kb
25,988 kb
25,990 kb
25,992 kb
25,994 kb
[0 - 143]
GRMZM2G139583_T02
GRMZM2G139583_T01

ZmVIII-3

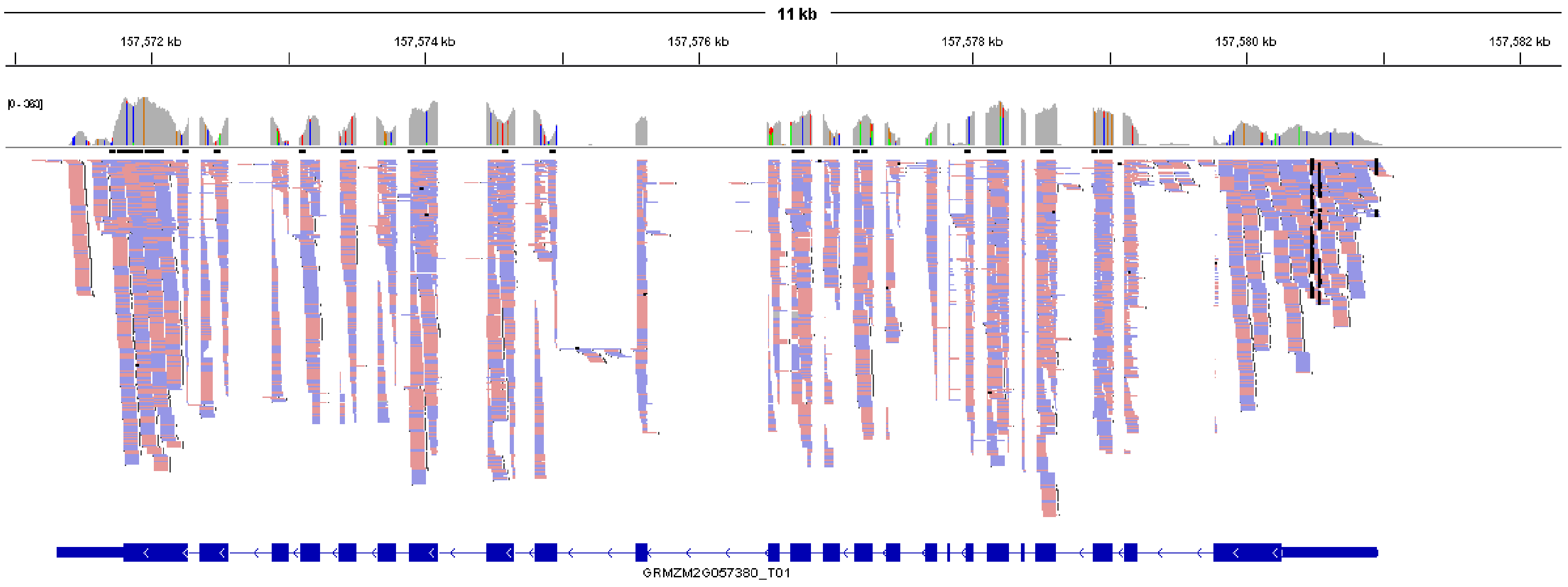

11 kb
157,572 kb
157,574 kb
157,576 kb
157,578 kb
157,580 kb
157,582 kb
[0 - 383]
GRMZM2G057380_T01

ZmXI-1

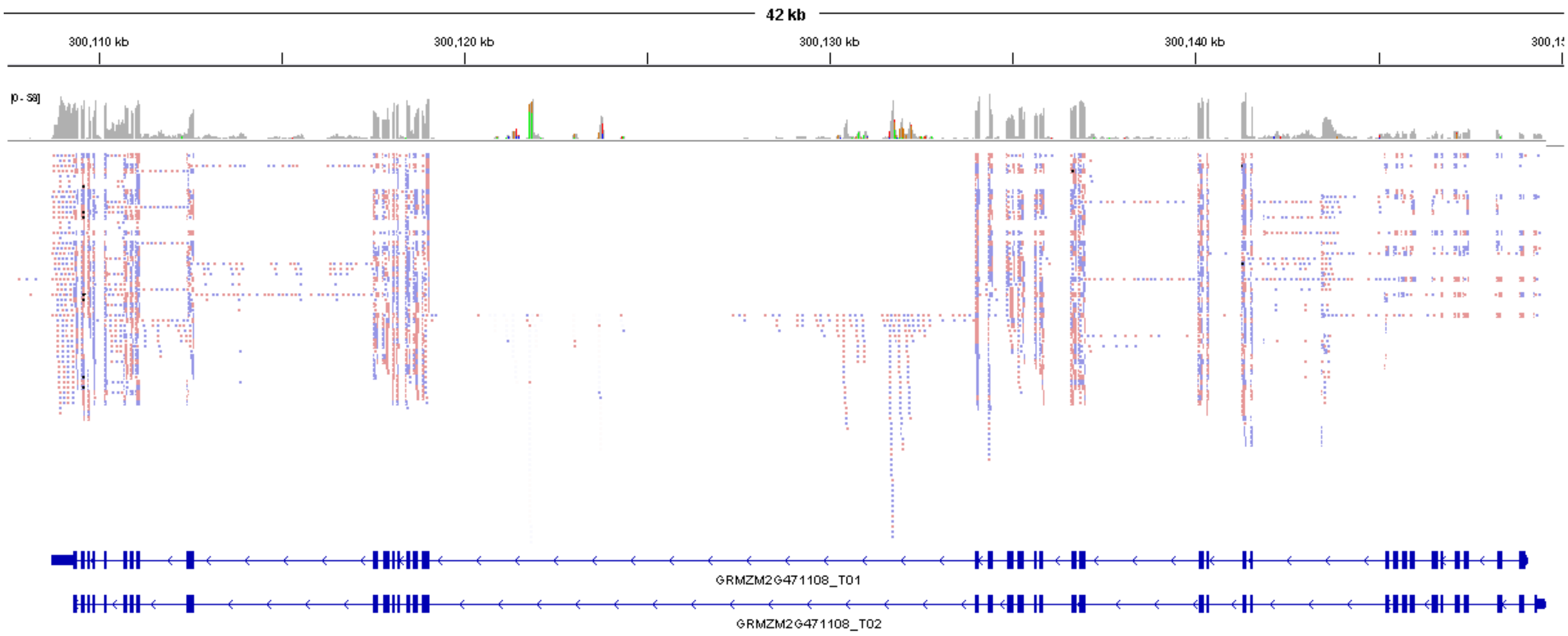

42 kb
300,110 kb
300,120 kb
300,130 kb
300,140 kb
[0 - 58]
GRMZM2G471108_T01
GRMZM2G471108_T02

ZmXI-3

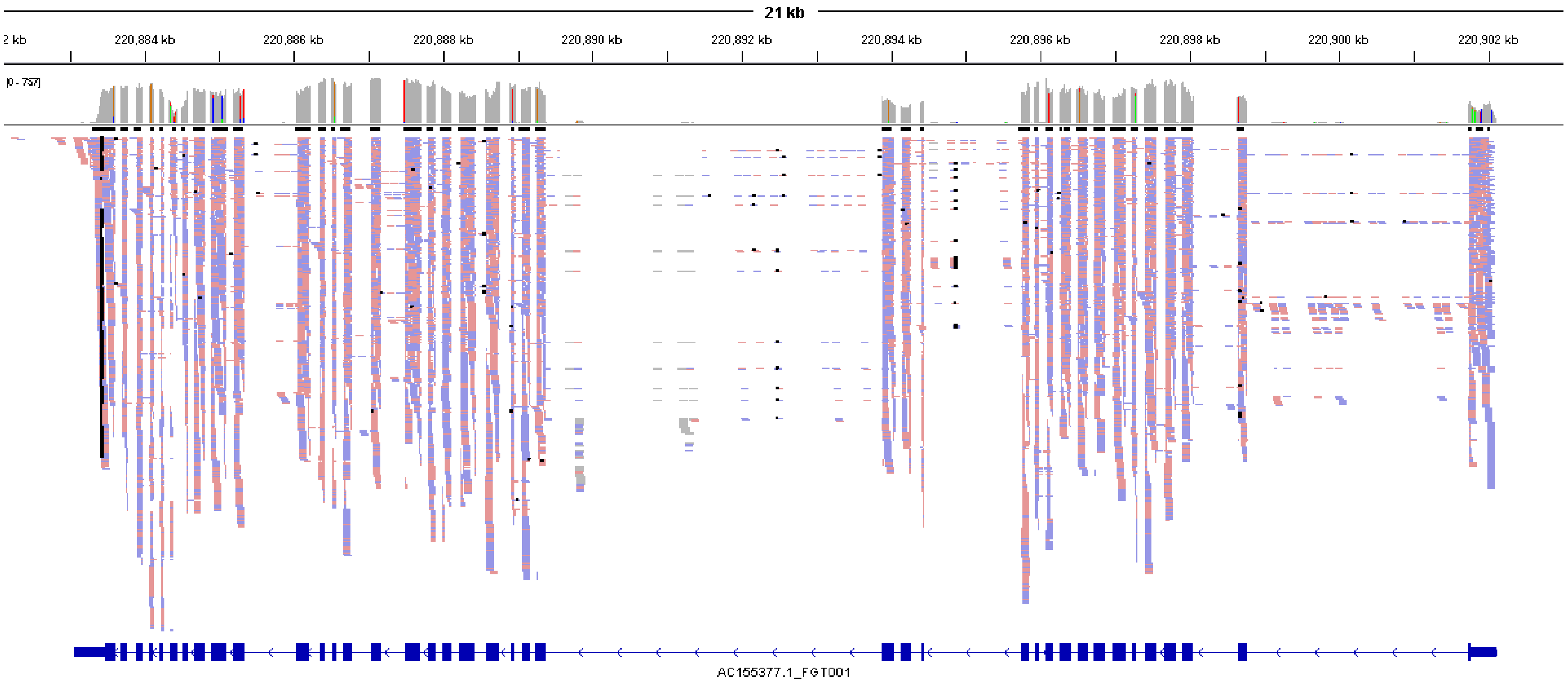

21 kb
2 kb
220,884 kb
220,886 kb
220,888 kb
220,890 kb
220,892 kb
220,894 kb
220,896 kb
220,898 kb
220,900 kb
220,902 kb
[0 - 757]
AC155377.1_FGT001

ZmXI-4

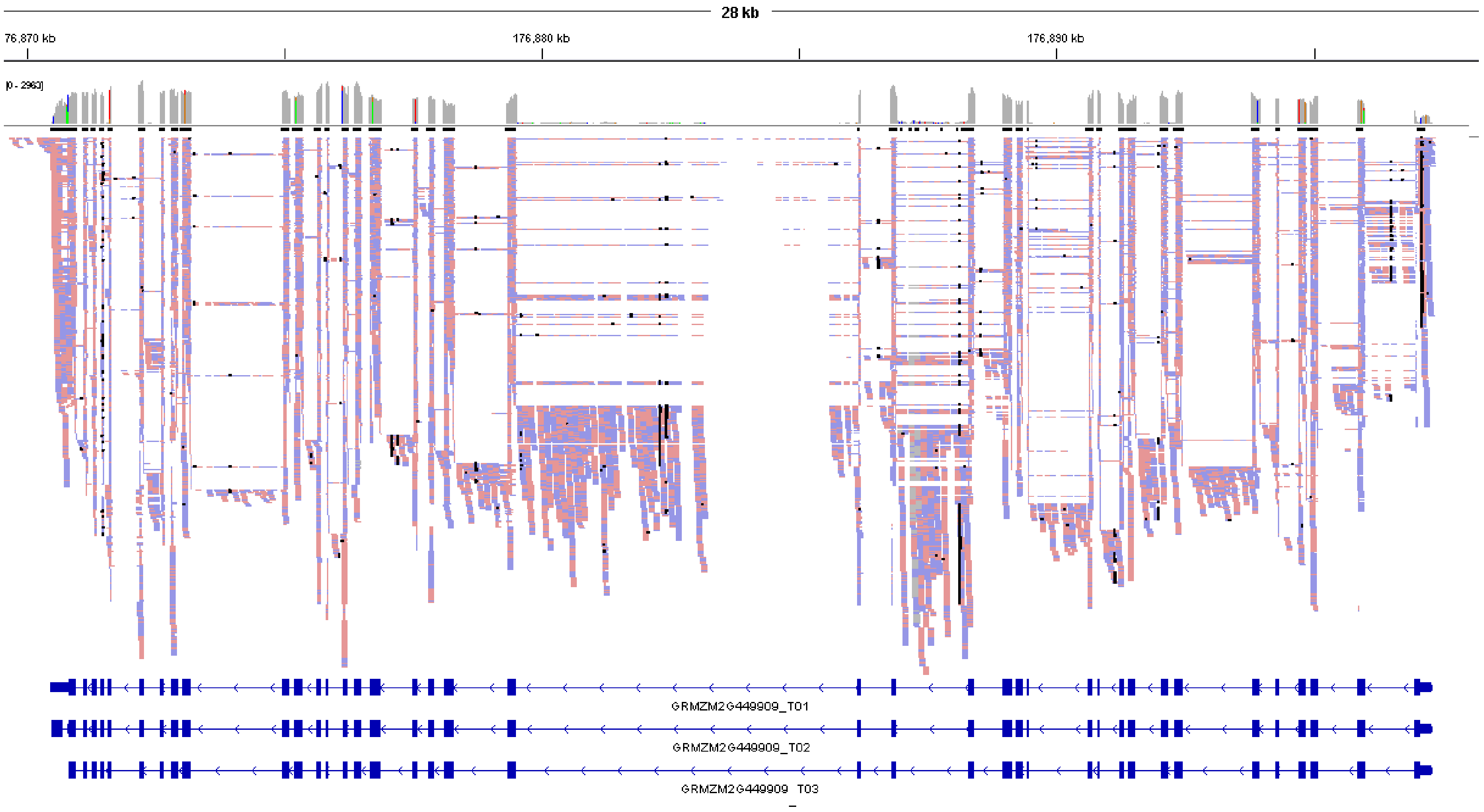
28 kb
76,870 kb
176,880 kb
176,890 kb
[0 - 2963]
GRMZM2G449909_T01
GRMZM2G449909_T02
GRMZM2G449909_T03

ZmXI-5

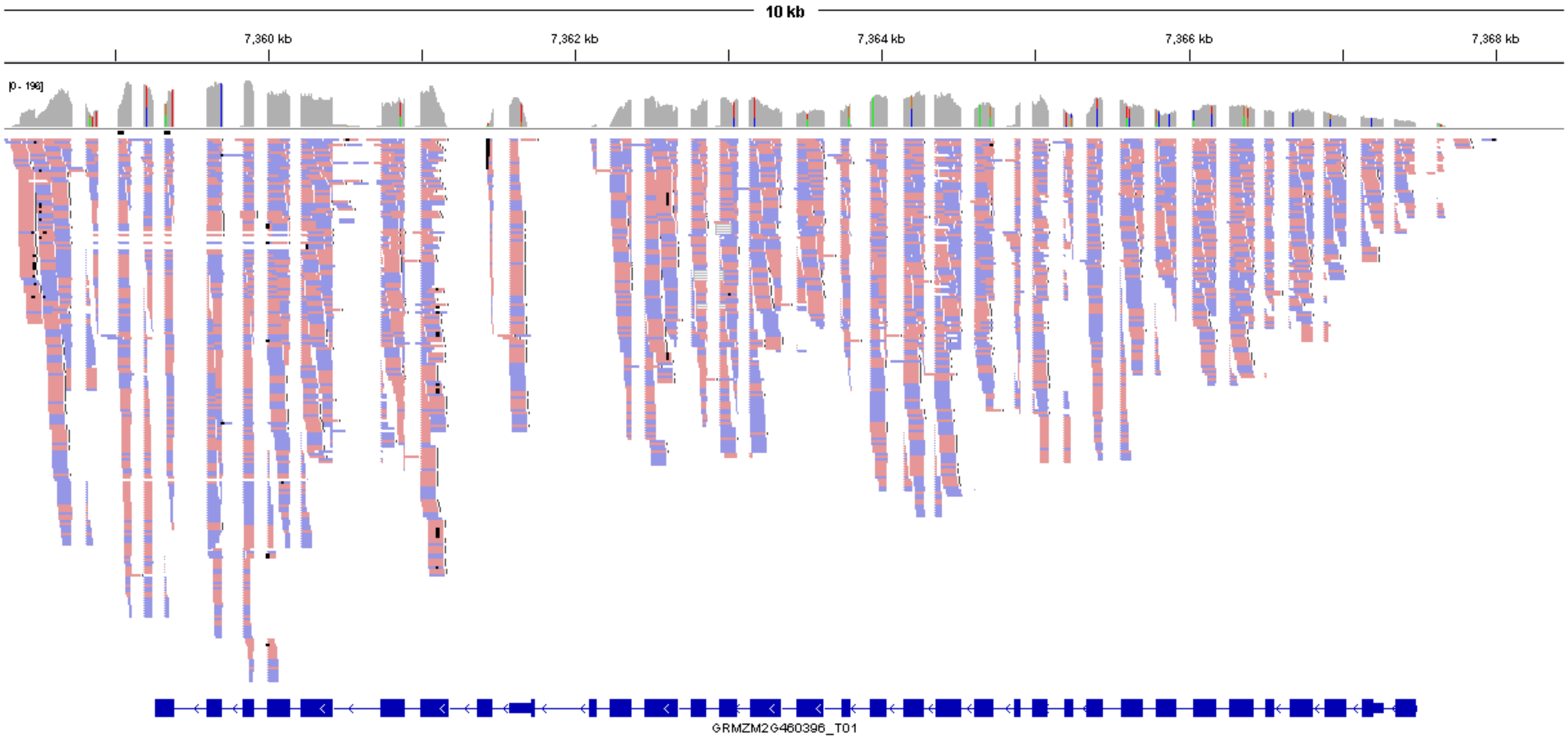

10 kb
7,360 kb
7,362 kb
7,364 kb
7,366 kb
7,368 kb
[0 - 196]
GRMZM2G460396_T01

ZmXI-6

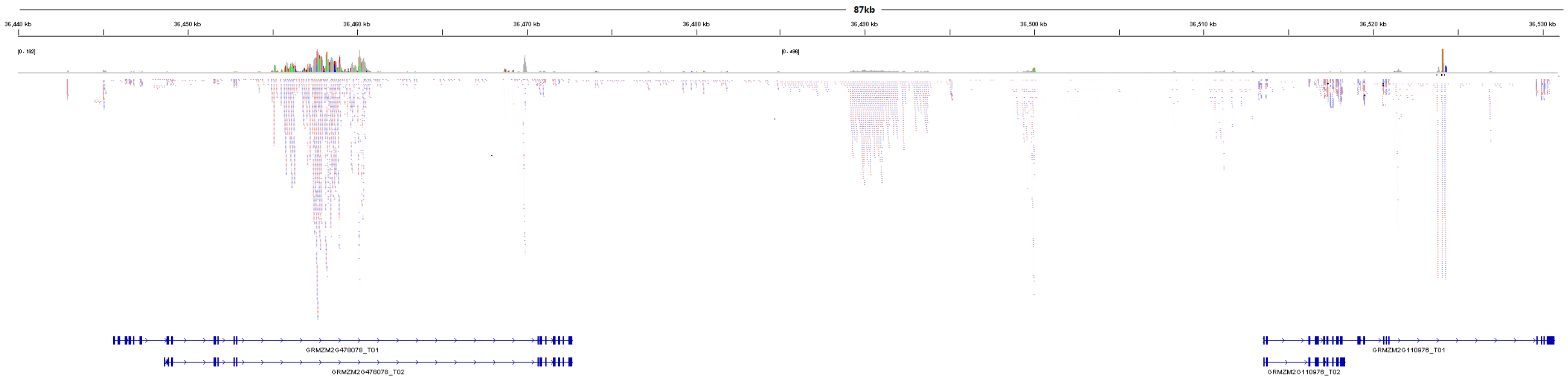
87kb
36,440 kb
36,450 kb
36,460 kb
36,470 kb
36,480 kb
36,490 kb
36,500 kb
36,510 kb
36,520 kb
36,530 kb
[0 - 182]
[0 - 496]
GRMZM2G478078_T01
GRMZM2G478078_T02
GRMZM2G110976_T01
GRMZM2G110976_T02

ZmXI-7

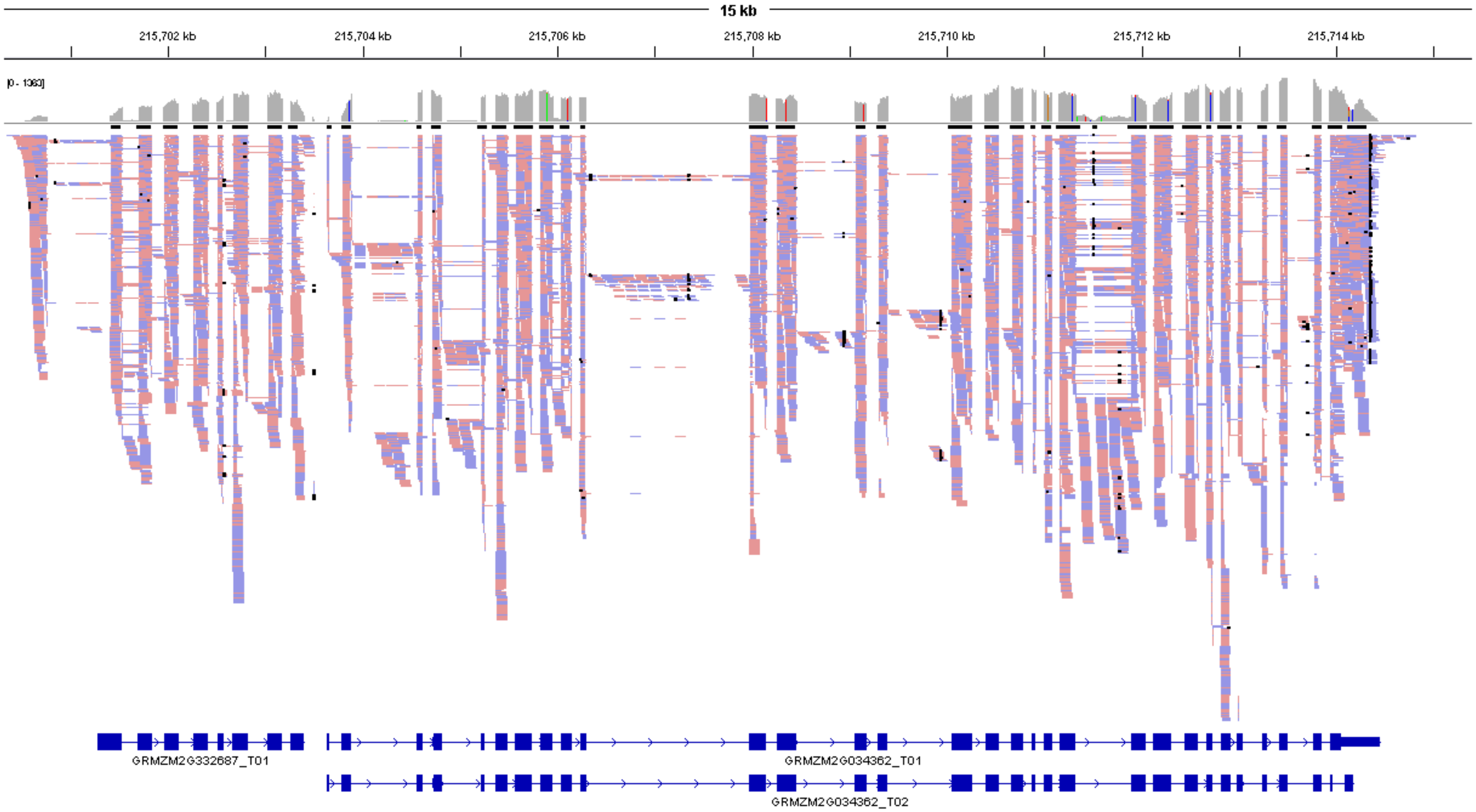
15 kb
215,702 kb
215,704 kb
215,706 kb
215,708 kb
215,710 kb
215,712 kb
215,714 kb
[0 - 1363]
GRMZM2G332687_T01
GRMZM2G034362_T01
GRMZM2G034362_T02

ZmXI-8

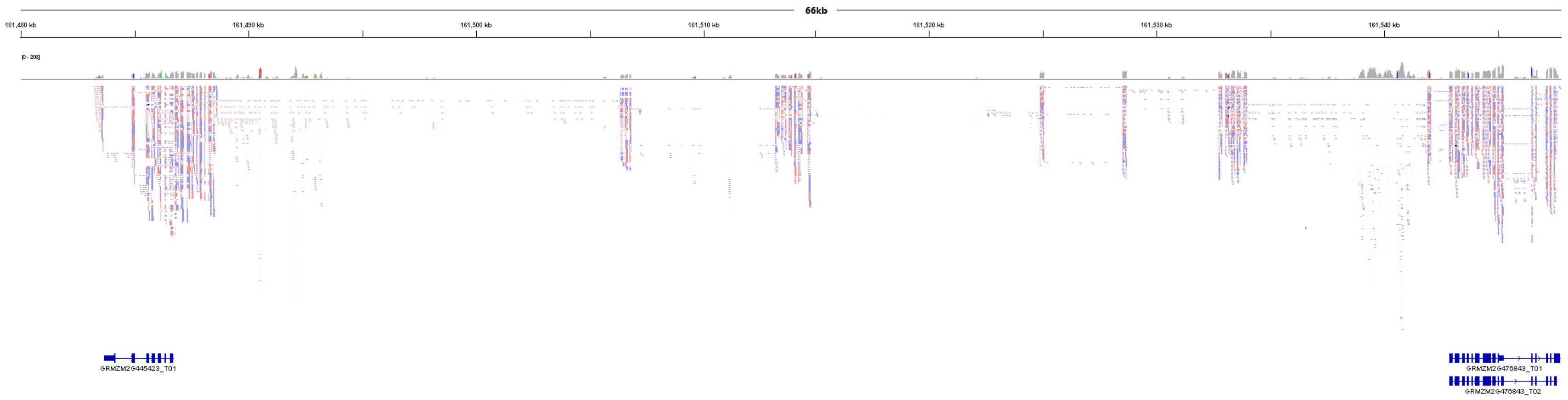

66kb
161,480 kb
161,490 kb
161,500 kb
161,510 kb
161,520 kb
161,530 kb
161,540 kb
[0 - 208]
GRMZM2G445423_T01
GRMZM2G476843_T01
GRMZM2G476843_T02

ZmXI-9

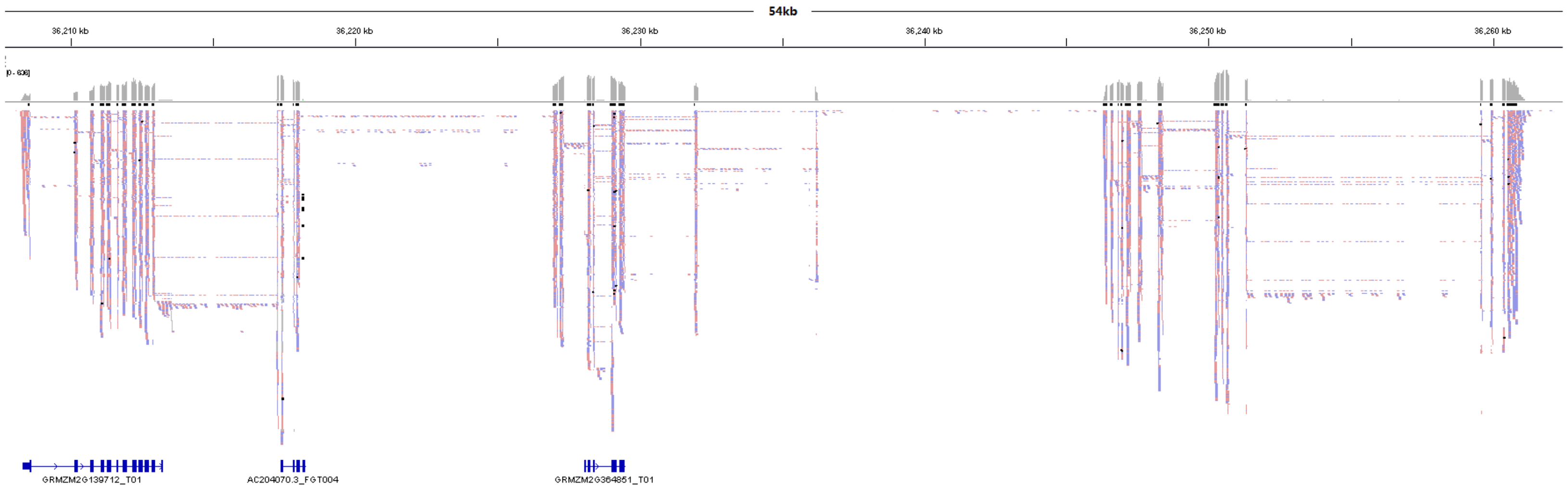

54kb
36,210 kb
36,220 kb
36,230 kb
36,240 kb
36,250 kb
36,260 kb
[0 - 636]
GRMZM2G139712_T01
AC204070.3_FGT004
GRMZM2G364851_T01

ZmXI-10

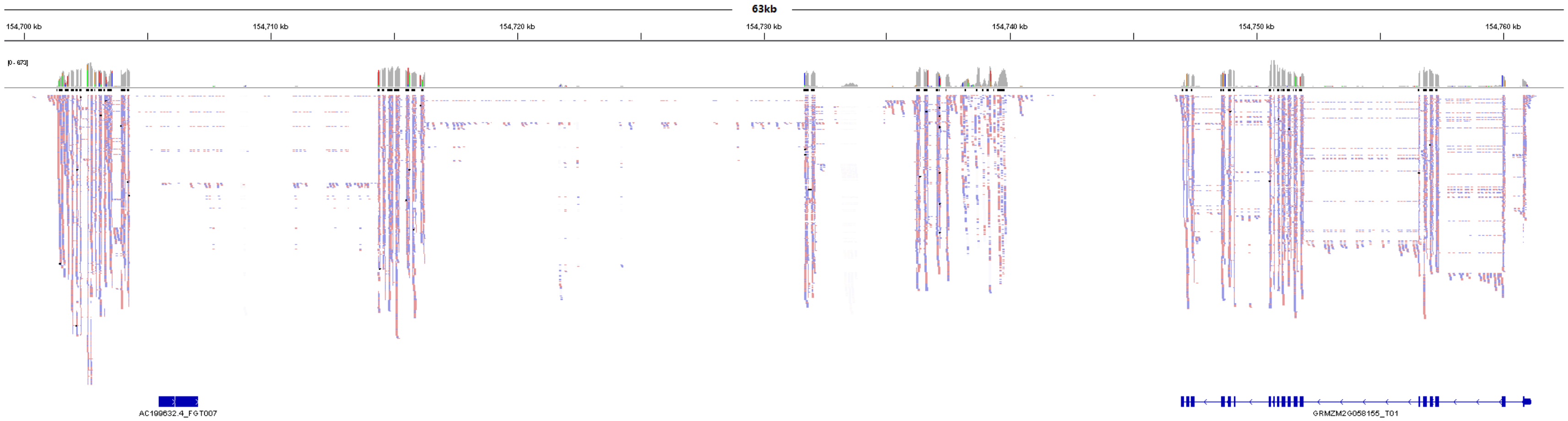

63kb
154,700 kb
154,710 kb
154,720 kb
154,730 kb
154,740 kb
154,750 kb
154,760 kb
[0 - 673]
AC199632.4_FGT007
GRMZM2G058155_T01

Supplementary Figure S3. The determination of the headless derivative of *O1* using two independent 5′ RACE experiments.

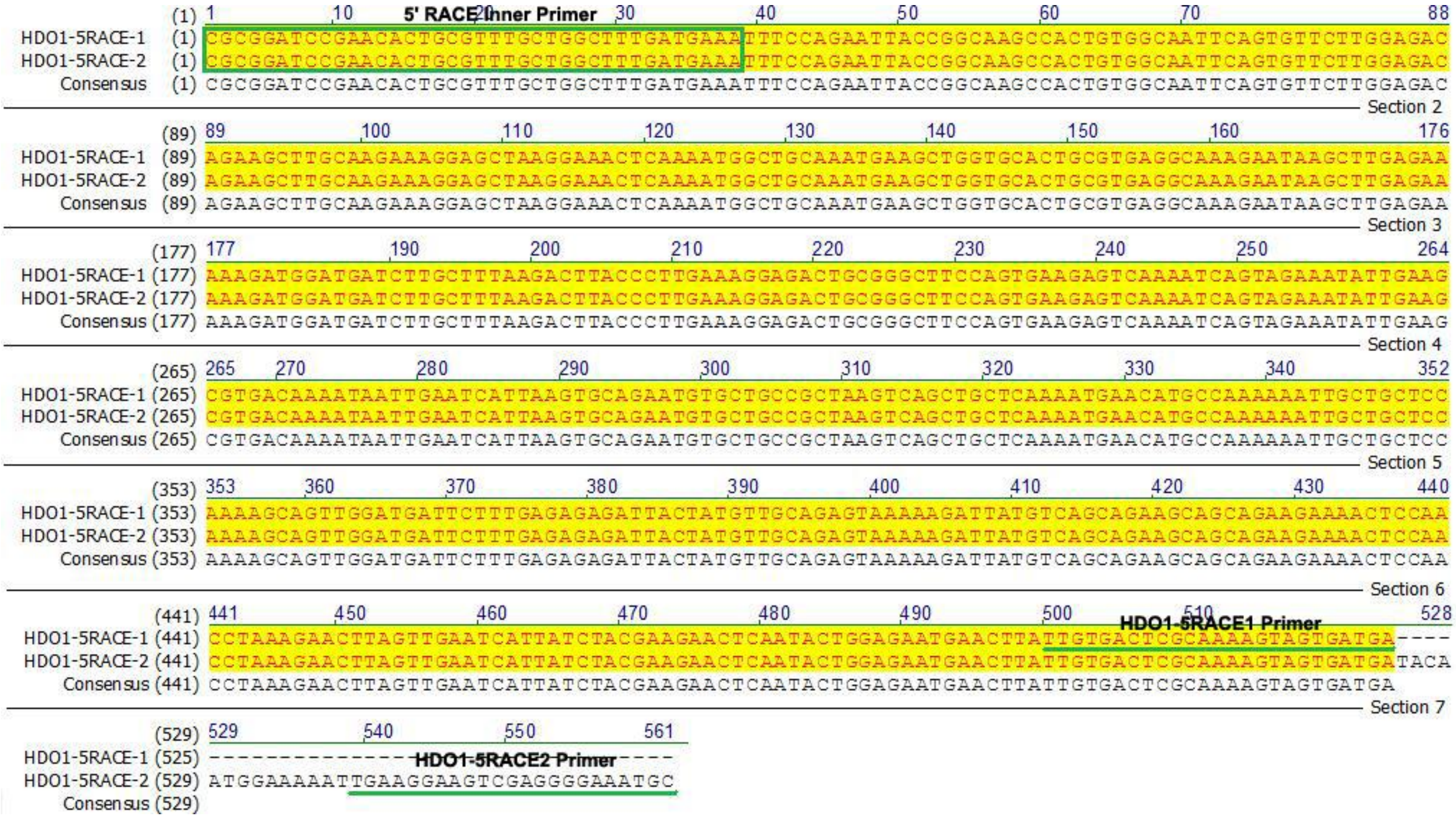

Supplement: Supplementary Data [file supp_ert437_jexbot107649_file001.pdf]
